# A longitudinal area classification of migration in Great Britain: Testing the application of Group‐Based Multi‐Trajectory Modelling

**DOI:** 10.1002/psp.2694

**Published:** 2023-07-14

**Authors:** Caroline Kienast‐von Einem, Jenna Panter, Alice Reid

**Affiliations:** ^1^ MRC Epidemiology Unit University of Cambridge Cambridge UK; ^2^ Department of Geography University of Cambridge Cambridge UK

**Keywords:** area classification, Group‐Based Multi‐Trajectory Modelling, longitudinal analysis, migration modelling, residential relocation

## Abstract

The migration of people affects the geographical distribution of the population and the demographic composition of areas over the short, medium and long terms. To recognise and respond to the corresponding needs and challenges, including consequences for service provision, social cohesion and population health, there is a continuing need to understand migration patterns of the past and present. Area classifications are a useful tool to simplify the inherently complex data on migration flows and characteristics. Yet, existing classifications often lack direct migration measures or focus solely on cross‐sectional data. This study addresses these limitations by employing Group‐Based Multi‐Trajectory Modelling (GBMTM) to create a longitudinal, migration‐specific classification of Great Britain's wards from 1981 to 2011, using six migration indicators. Using U.K. census data, we reveal six distinct migration clusters that highlight the rapid growth in studentifying neighbourhoods, the continuous influx of migrants into inner cities, and a noticeable North–South divide in terms of movers' tenure enforced by persisting income selectivity. Additionally, the geographical distribution of clusters shows a common pattern in urban areas irrespective of size or location. The longitudinal perspective of our GBMTM classification highlights trends and changes in migration patterns that are not well reflected in either the general purpose or the cross‐sectional migration classification that we used as comparators. We conclude that the method presented and the classification generated offer a novel lens on migration and provide new opportunities to explore the effects of migration on a variety of outcomes and at various scales.

## 1 INTRODUCTION

In the year to mid‐2020 around 622,000 people moved to the United Kingdom, 375,000 people left the United Kingdom, and 3.2 million people moved between local authorities within the United Kingdom (The Office for National Statistics., 2021). Both international and internal migration make major contributions to population change by affecting the size and composition of local populations (T. Champion, 2005; T. Champion & Shuttleworth, 2017b; Lomax et al., 2014; Stillwell & Dennett, 2008; UK Statistics Authority, 2009). For example, many parts of London are growing in population but, perhaps more interestingly, have been simultaneously experiencing an influx of young movers and losing many older adults to the rest of the United Kingdom (Champion, 2005; Lomax et al., 2014). Different migration flows can have profound consequences for service provision and labour supply (Poppleton et al., 2013; Rees et al., 2013; Travers et al., 2007), social cohesion and crime (Bailey et al., 2012; Bell & Machin, 2011; Phillips & Robinson, 2015; Rotolo & Tittle, 2006), the physical environment (Bernt, 2019; Collins et al., 2020; Datta, 2009; Hogan, 1992), economic development (Lisenkova et al., 2014), and population health (Brown & Leyland, 2009; Darlington et al., 2015; Higgins et al., 2019; Wallace & Kulu, 2014). To guide government policy, enable efficient resource allocation, and understand the general and local challenges of Britain's population, a thorough understanding of migration is essential.

The influence of migration on the local population can be divided into (a) the number of migrants (which affects the population size) and (b) the types of migrants (which influences the population composition). A substantial body of research has explored the number of migrants and variations across space and time. As early as 1885, Ravenstein (1885, 1889) found that urban areas in the British Isles gained more migrants than rural areas, attributing their population growth to in‐migration rather than natural increase through births. Later, Willatts and Newson (1953) and Webb (1963) expanded these ideas by classifying areas based on migrant numbers and total population change. Mapping the resulting ‘migration types’ indicated significant urbanisation between the 1920s and 1950s and increased net migration into London and the South‐East of England. In the following decades, the relationship between urban areas and net migration changed, with most U.K. cities experiencing a noticeable decline in inward migration (Champion, 1989; Cross, 1990; Kennett, 1979; Rees et al., 1996) and more complex patterns of spatial mobility emerging from the late 1980s onwards (Owen & Green, 1992; Stillwell et al., 1992). Beyond the mere reporting and monitoring of changes in population sizes, which remains a primary research focus, a small number of studies have explored its impact on the populations undergoing change, revealing interesting links to economic efficiency, social capital, well‐being, and political participation (Akarca & Tansel, 2015; Bernt, 2019; Champion, 1999; Cooke, 2011; Crowder & South, 2005; Kaplan & Schulhofer‐Wohl, 2012; Lisenkova et al., 2014; van Dalen & Henkens, 2011).

In addition to affecting the size of local populations, migration influences their composition. Migrants differ from the general population in terms of age, life stage, health, housing tenure, socioeconomic position and educational achievement (Boyle et al., 1998, 2001; Buck et al., 1994; Champion et al., 1998; Livingston & Bailey, 2007). The link to age and life course has been particularly emphasised, with young adults being more likely than any other age group to have moved within the country in the previous year (Bailey & Livingston, 2005; Beatty et al., 2009; Champion, 2005; Clark, 2013). Additionally, migrants differ from each other. As early as the mid‐nineteenth century, Farr (1864) had concluded that migrants from urban to rural areas differed in their health from those moving from rural to urban areas. Later, Welton (1872), Hill (1925), and Bentham (1986) found similar results. That migrants are different from the people they leave behind and the people they end up with indicates that migration is selective, and this in turn emphasises the role of place characteristics in ‘pushing’ or ‘pulling’ different types of movers into specific locations (Law & Warnes, 1976 and Rogers Walters, 2000). Overall, the distinctiveness of migrants compared to the general population, their differences among themselves and in their destination preferences, highlight how migration can change the character of local populations over time, potentially reinforcing regional inequalities (Norman et al., 2005).

Modelling migration is inherently difficult: A person can move an infinite number times in their lifetime (Lomax et al., 2013), and each move has effects on at least two dimensions, the origin and the destination location (Dennett & Stillwell, 2011). Additionally, depending on who is arriving and who is leaving, the effects of a move on an area and its local population can vary drastically. To simplify the complexity of migration data, some authors have turned to classification techniques which group together areas with similar characteristics before undertaking further analysis (Dennett & Stillwell, 2010; Raymer & Giulietti, 2009; Rees et al., 1996). Area classifications provide an easily interpretable overview of the spatial distribution of demographic and socioeconomic characteristics, and this can help identify regional differences and factors that influence migration decisions (Webber & Craig, 1978). Vickers (2006) argues that it is not possible for the human mind to simultaneously process information about the individual moving events of every member of a population, and that area classifications present a convenient technique for addressing this issue. Such classifications organise large data sets so as to enhance understanding and the efficient retrieval of information. While other methods of analysing migration can provide more detailed insights into the experiences of individual migrants or of one isolated migration measure, area classifications provide a valuable summary of migration patterns across different regions. This allows policymakers and researchers to, for instance, identify which areas are attracting or repelling movers and explore the underlying reasons and consequences behind these patterns.

To date, the majority of existing area classifications in the United Kingdom are based on the total population and do not include specific migration variables (e.g.: Office for National Statistics, 2018; Vickers et al., 2001). Whilst such classifications can be helpful to, for example, simplify the range of origins or destinations for ease of analysis or summarise the stock of foreign‐born residents, they tell us little about the direct effect migration has on these figures and the movement of locals. Additionally, analyses by Dennett and Stillwell (2011) and Duke‐Williams (2010) show noticeable differences between the overall and the internal migrant populations across multiple variable domains and different spatial scales. In such cases, analysing migration data through cluster groups defined from the general population could obscure or distort migration patterns. Dennett and Stillwell (2011) make a compelling case for a migration‐specific classification to identify important differences in migration patterns and effects, and offer a new, potentially more useful lens through which to examine migration.

To our knowledge, there is only one existing example of a migration‐specific classification in Great Britain, the Centre for Interaction Data Estimation and Research (CIDER) classification (Dennett & Stillwell, 2011). The CIDER classification considers cross‐sectional data from the year 2001 and focuses on internal migration in the United Kingdom. The main advantages of the CIDER classification include its ability to capture a wide range of social and economic characteristics of the migrant population and offer a robust tool for classifying and comparing small geographical areas. However, the reliance on cross‐sectional data limits conclusions about patterns, trends, and factors that contribute to migration over time (Willatts & Newson, 1953). Re‐evaluating cross‐sectional versus longitudinal models of residential mobility, Clark (1992), for instance, confirmed that longitudinal models reveal additional complexities not obvious in cross‐sectional analysis of mobility and migration. Due to methodological limitations, area classifications or cluster analyses of migration thus far tend to be either multi‐variate but cross‐sectional (such as the CIDER classification for the United Kingdom and classifications by Bernard et al., 2017 for Latin America, and Škuflić et al., 2018 for the European Union), or longitudinal but univariate (such as classifications by Akarca and Tansel, 2015 for Turkey and Ovchinnikova 2016 for the Khmelnytskyi region in the Ukraine). To date, there is no migration‐centred area classification that simultaneously incorporates multiple variables and their progression over time.

In this paper, we use Group‐Based Multi‐Trajectory Modelling (GBMTM) to develop a new migration classification for Great Britain that is multi‐variate and longitudinal. GBMTM is a clustering technique identifying latent groups following similar developments in multiple outcomes over time (Nagin, 2005). Thus far, the method has found wide application in criminology and clinical medicine but has mainly been applied to individual level data. A Scopus literature review reveals that between 2012 and 2022 the number of studies employing GBMTM or its univariate adaptation GBTM increased from 9 to 265 publications per year. Examples range from Girard et al. (2019) exploring the origins and development of aggression subtypes in children, through Zhuang et al. (2022) investigating patterns and predictors of healthcare utilisation among palliative cancer patients, to Magrini (2022a) comparing the agricultural sustainability in European Union countries (one of the first area‐level applications of GBMTM). These diverse examples demonstrated a shared focus on the longitudinal development of multiple variables and how these contribute to the formation homogeneous groups, along with corresponding analyses of group effects and needs. By extending GBMTM to aggregate area‐level data, we aim to develop a new migration classification that presents a more complete picture of migration in Great Britain over the last 40 years while simultaneously testing the method's utility for migration analyses. To this end, this paper has two principal research questions: Firstly, does GBMTM lend itself to an area‐level application of longitudinal migration data in the United Kingdom, and what are the methodological challenges for such application? Secondly, what migration clusters are revealed through the analysis and what do they tell us about the migration landscape of Great Britain? Notably, the focus of this classification is on migration flows in the few years before each census rather than numbers of people not born in the place they were living, meaning it reflects recent migration events at different time periods rather than migrants who moved and remained in place.

## 2 METHODS

### 2.1 Data source

The study utilised aggregated, small‐area level data collected in the 1981, 1991, 2001, and 2011 U.K. Censuses. The Census is a questionnaire survey of individuals in all households in the country, carried out every 10 years. With response rates around 98% (Office for National Statistics 2016a, 2016b, 2016c; Simpson & Dorling, 1994), the Censuses provide the most representative data for the entire U.K. population. The 2021 census (and 2022 census of Scotland) results were not included in the classification as data were not consistently available for all GB nations at the time of writing, and concern about the distorting effect of the Covid‐19 pandemic on migration patterns (Lomax, 2022; Nanda et al., 2021).

Data collected through the U.K. Censuses are released in a number of forms. Between 1981 and 2011 these datasets have varied in number, scale and focus. The ‘Special Migration Statistics’ (SMS) data release contains most of the migration data for the 1981 and 1991 censuses (Duke‐Williams, 2017). For 2001 and 2011, this data set was remodelled to ‘Origin Destination’ data or ‘flow’ data (Rees et al., 2002). Additionally, some migration information can be found in the general data set releases, such as the Small Area Statistics (SAS) in 1981, the SAS and the Local Base Statistics (LBS) in 1991, and up to seven different data tables from 2001 onwards (Rees et al., 2002).

### 2.2 Area boundaries

To allow comparison through time, data were needed for areas with a consistent set of boundaries over time. In practice, area boundaries are subject to continuous change leading to the creation of new administrative areas and the splitting, merging or abolition of others. The UK Data Service's portal for flow data ‘WICID’ provides 1981 and 1991 SMS data pre‐converted into standard table (ST) ward boundaries in 2001. The extensive procedure for this conversion is detailed in Boyle and Feng (2002) and Feng and Boyle (2010). To utilise this high‐quality, pre‐converted data and, as far as possible, avoid problems with changing boundaries over time, 2001 ST wards were chosen as the units of analysis. ST wards include a minimum of 400 households and 1000 persons (Office for National Statistics, 2012). They are considered appropriate for work on both population change and migration (Rees et al., 1996). In total, 9976 ST wards were included in the analysis: 8800 in England and Wales and 1176 in Scotland. Northern Ireland was omitted from the analysis due to difficulties in converting output boundaries into ST wards and lack of variable comparability.

1981 and 1991 variables that could not be accessed through WICID, and 2011 data that were not available ‘pre‐converted’ into 2001 ST wards, were manually adapted. For these conversions, different translation tables were utilised depending on the year of the original data and the national background. If conversion tables provided proportions, data were split proportionally following the most common areal interpolation technique: ‘areal weighting’. Despite valid objections to the utility of this approach in migration studies, based on the facts that migration decays with distance and populations are not evenly distributed across areas (Boyle & Feng, 2002), it remains a reasonable solution in the absence of more granular ancillary data (Tapp, 2010; Xie, 1995). In cases where no proportions were provided, data were divided equally. All manual conversions were first tested on data for which Boyle and Feng's conversions were available and only utilised if they did not result in significantly different output. An overview of the manual conversions and their validation is attached in Supporting Information: Appendix 1.

### 2.3 Migration

In the U.K. Census, migrants are identified as those whose address at the time of enumeration is different from that 1 year previously (‘One year ago, what was your usual address?’). The question was asked at all four time points and intends to identify all those moving within the United Kingdom and to the United Kingdom from abroad. People who emigrate to a different country do not receive a census questionnaire and are thus not captured. The current study achieves a sample size of 5,037,878 (1981), 4,965,040 (1991), 7,075,844 (2001) and 6,760,198 (2011) movers, between 9% and 11% of the GB population.

Although the Census is one of the most extensive and reliable sources of migration data for the United Kingdom (Lomax, 2022), it does not capture all moves (Bailey & Livingston, 2005). For example, people who moved away from an address and then moved back within the same year are not recorded as migrants. Similarly, people who moved multiple times in the past year are documented in the same way as those who moved once, and those who moved just over a year ago are not identified at all. In the current study, some additional simplifications were made to ensure comparability between the four censuses. Firstly, only main addresses were used to classify moves; secondly, internal short‐distances moves were not distinguished from internal long‐distance ones.

### 2.4 Migration indicators

The selection of variables is principal to the quality and validity of any clustering method. Variables were included if they provided conceptually meaningful information and showed (a) consistency in measurement across geographies, (b) absence of high correlation with other included variables, and (c) variation over space. Detailed principles of variable selection in cluster analysis for area classification are described in Everitt et al. (2001), Milligan (1996) and Vickers et al. (2001).

The GBMTM application in Stata and SAS limits the number of indicators to six, so we included two indictors that capture the percentage of movers (international and internal) in the total population, and four that describe the characteristics of movers in terms of age and tenure (Table 1). As migration is linked to life‐course events, age is helpful in explaining moving motives; for example it can help to distinguish a student mover starting university from a retiree locating towards the seaside. As a change in tenure accompanies each move, it is a good reflection of a mover's socioeconomic status at the time of moving. Additionally, it provides information on the area's housing stock. To avoid bias in the selection of variables, several sensitivity analyses were performed by running alternative multi‐variate models with just five indicators or by substituting one of the six indicators with a different variable.

**Table 1:** Migration indicators.

Indicator	Calculation	1981	1991	2001	2011	Adjustment for missing/incomplete origin addresses?
The percentage of internal in‐movers amongst residents	$\frac{{\text{Internal inmovers}}^{\$}}{{\text{Usual residents}}^{*}}\times 100$	✓	✓	✓	✓	✓
The percentage of international in‐movers amongst residents	$\frac{{\text{International inmovers}}^{£}}{\text{Usual residents}}\times 100$	✓	✓	✓	✓	✗
The percentage of in‐movers aged 18–24 years	$\frac{\text{Internal Inmovers aged}\;18\;\text{to}\;24\;\;+\;\text{International inmovers aged}\;18\;\text{to}\;24\;}{\text{Internal inmovers}\;+\;\text{International inmovers}}\times 100$		✓	✓	✓	✓
The percentage of in‐movers aged 65 years or over	$\frac{\text{Internal Inmovers aged}\;65\;\text{and over}+\text{International Inmovers aged}\;65\;\text{and over}}{\text{Internal Inmovers}+\text{International Inmovers}}*100$		✓	✓	✓	✓
The percentage of in‐moving households who owner occupy¥	$\frac{\text{Internal inmover households owner occupying}+\;\text{International inmover households owner occupying}}{\text{Internal inmover households}+\;\text{International inmover households}}\times 100$		✓	✓	✓	✓ (not for 1991)
The percentage of in‐moving households who live in Social Housing†	$\frac{\text{Internal inmover households in social housing}+\;\text{International inmover households in social housing}}{\text{Internal inmover households}+\;\text{International inmover housholds}}\times 100$		✓	✓	✓	✓ (not for 1991)

*Note*: Overview of the indicators included in the Group‐Based Multi‐Trajectory Model: the selected migration indicators, their underlying calculations, and which years they are available for, identifying the indicators available to include in an adjustment for missing/incomplete origin addresses.

^$^Internal in‐movers includes those aged 1 year or over who indicated that they live in a ward at the time of enumeration but resided at a different address within Great Britain1 year previously, including addresses within the same ward.

*Usual residents refers to all residents of a ward/the population base at the time of enumeration.

^£^International in‐movers includes those aged 1 year or over who indicated that they live in a ward at the time of enumeration but resided at a different address outside of Great Britain 1 year previously.

^¥^ In‐moving households are households whose household head indicated that they live in a ward at the time of enumeration but resided at a different address in, or outside of, Great Britain 1 year previously. Owner‐occupation is determined as households which own their accommodation outright, with a mortgage or loan, or under shared ownership.

^†^ In‐moving households are households whose household head indicated that they live in a ward at the time of enumeration but resided at a different address in, or outside of, Great Britain 1 year previously. ‘Living in Social Housing’ refers to households renting from a housing association, council, local authority, New Town, or Scottish Homes.

### 2.5 Analysis

#### 2.5.1 Model selection

GBMTM is, in its simplest form, a clustering technique able to identify latent groups following similar developments in multiple outcomes over time (Nagin, 2005). Through several regression and Maximum Likelihood calculations, the model approximates the shape the different longitudinal trajectories present in the data and the probability of items following these trajectory patterns (Nagin, 2005). To classify the 9976 wards according to their trajectories across the six migration indicators, several models had to be fitted and evaluated. A detailed description of the selection process and the analytic method is attached in Supporting Information: Appendix 1. Three versions of software are available for estimating the multi‐trajectory models, operating on SAS, Stata, and R. All three software plug‐ins can be freely downloaded. For this study, we utilised Stata version 16.

#### 2.5.2 Interpretation and validation

Latent class profiling is a common procedure for model validation (Klijn et al., 2017). Taking a classify‐analyse approach, the generated clusters were named, tabulated on different characteristics and mapped. Additionally, we use ANOVA to explore statistical differences between established groups across several variables.

Beyond latent class profiling, the results were compared to one general‐purpose classification from the Office for National Statistics (ONS) and to the CIDER migration classification. The ONS classification is produced using k‐means clustering and consists of a three‐tiered classification into super‐groups, groups and subgroups for local authorities and output areas (Gale et al., 2016). It is based on 2011 census data covering five domains: demographic structure, household composition, housing, socioeconomic character, and employment (Office for National Statistics, 2018). The CIDER migration classification (Dennett & Stillwell, 2011) also used k‐means clustering and presents eight migration groups based on 56 variables from the 2001 Census Special Migration Statistics. The clustering units are local authority districts. Neither classification encompasses data from multiple time points. To allow comparisons with the new classification, both comparators were transformed to the 2001 output area level through areal weighting. The comparability between the different classifications was established using chi‐square tests of independence and Cramer's V. Additionally, the classifications were cross‐tabulated to explore groups of exceptionally strong or weak correspondence.

## 3 RESULTS

### 3.1 Class enumeration and model fit

Models with 2–7 groups were tested on their model fit and output. The fit indices for each model are presented in Table 2. A six‐group model emerged as the preferred option with well‐performing fit indices and, compared to the five‐group model, an additional group that showed unique characteristics. The ‘levelling out’ of the Bayesian Information Criterion (BIC) and the Akaike's Information Criteria (AIC) from the 5 to the 6 class models further indicated that the ideal model fit had been reached (sometimes described as an ‘elbow’ in the curve; Klijn et al., 2017). Adding a seventh group did not contribute any meaningful additional insights but largely duplicated existing groups. After deciding on the six‐group model, the polynomial terms were adjusted to fit each trajectory. The fully adjusted model resulted in a slightly better BIC and AIC (rising to −561642.07 and −561060.89, respectively) compared to the model with unadjusted, maximum polynomial orders.

**Table 2:** Fit indices for the tested Group‐Based Multi‐Trajectory Models.

Groups	BIC (Obvs)	AIC	Entropy	APPA	OCC	Mismatch^a^	Smallest group^b^
2	−592523.91	−592284.30	0.989	0.997928	44.61213	−0.0005749	8.42
				0.9842919	676.4717	0.0005749	
3	−586495.75	−586149.08	0.988	0.9969933	41.67628	−0.0003856	1.47
				0.9748463	361.4487	0.0003078	
				0.9956678	15285.23	0.0000778	
4	−571725.40	−571271.67	0.965	0.9862832	33.70537	−0.000488	1.77
				0.9628509	83.93561	0.000571	
				0.980327	710.9683	−0.00059	
				0.9852313	3698.312	−0.00024	
5	−564242.80	−563682.01	0.963	0.9841976	36.09532	−0.0001281	1.38
				0.9632009	96.14639	0.0000695	
				0.9518254	152.2349	0.0002008	
				0.9879372	3304.177	−0.0001713	
				0.992283	9147.161	0.0000292	
6^c^	−561646.65	−560978.80	0.967	0.9845982	37.22547	0.000072	0.50
				0.9643457	100.0744	0.0005564	
				0.9475669	150.0891	−0.0004911	
				0.9778698	2300.956	0.000097	
				0.9862717	2932.045	−0.0002415	
				0.9999979	95000000	0.0000073	
7	−558587.07	−557812.16	0.960	0.9783683	32.29247	−0.0017686	1.19
				0.9468889	643.044	0.0004131	
				0.9634981	101.1541	−0.0001518	
				0.9768429	1141.772	0.0001439	
				0.9320972	98.03004	0.0013368	
				0.995222	17251.6	0.0000011	
				0.9979832	39885.35	0.0000252	

*Note*: Overview of fit indices for the tested Group‐Based Multi‐Trajectory Models.

^a^ The mismatch between the proportion assigned to the group and the estimated probability of group membership.

^b^ The percentage of individuals estimated to be assigned to the smallest group.

^c^ The chosen model.

### 3.2 Cluster characteristics

**Figure 1:**
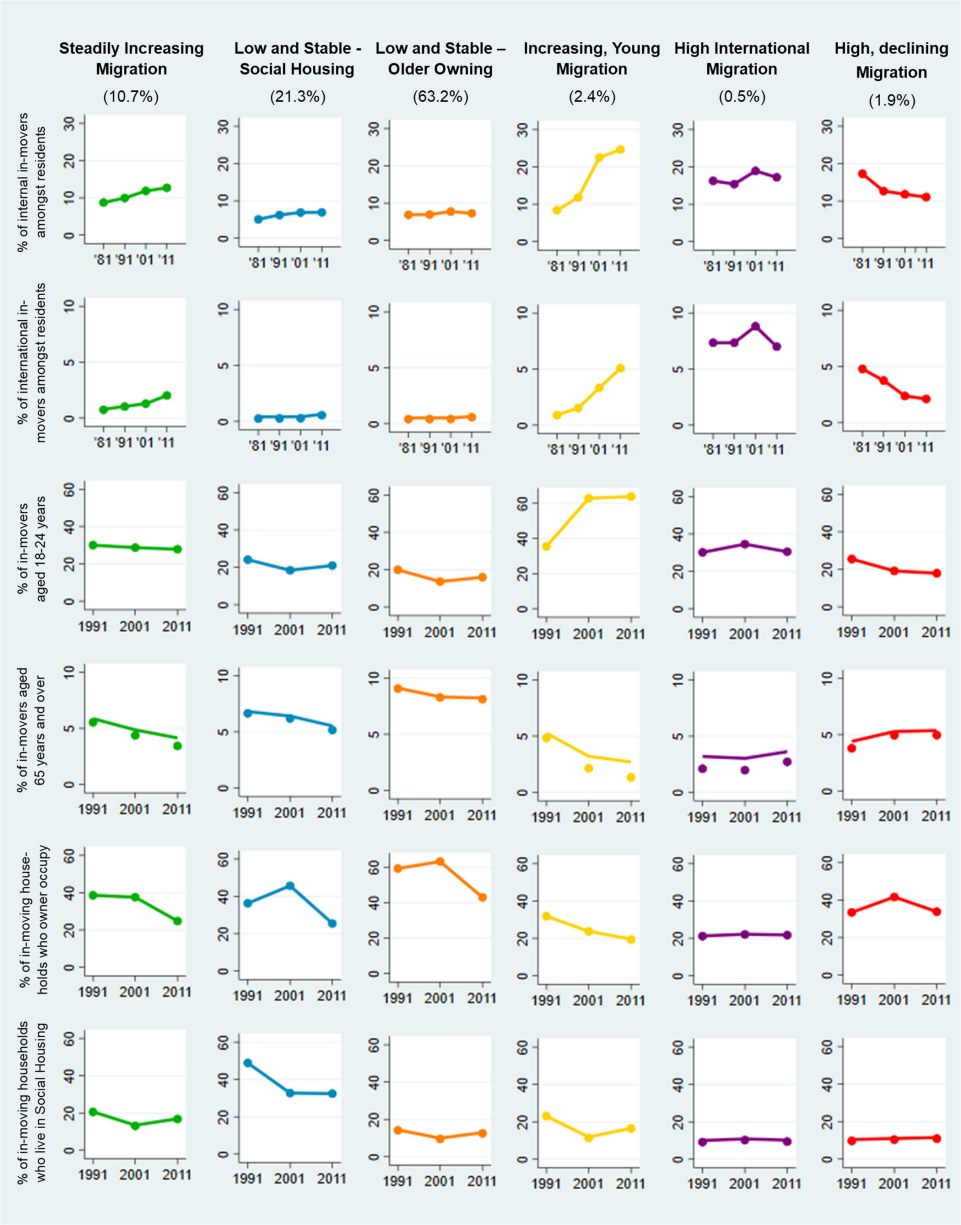
Group‐Based Multi‐Trajectory Model output. The output of the final six‐group multi‐trajectory model. The indicators are listed horizontally, and trajectories for each group are presented vertically beneath their title and percentage of the sample they account for. The graphs show mean values of the corresponding group.

The final Group‐Based Multi‐Trajectory Model revealed six distinct migration clusters. An overview of all clusters and their respective trajectories across the six migration indicators is provided in Figure 1. Additional, descriptive information is provided for 2011 (as an example) in Table 3.

To summarise, Cluster 1 contains areas with a moderate and slowly increasing proportion of internal and international in‐movers, we call it the ‘*Steadily Increasing Migration*’ cluster. Wards in Cluster 2 exhibit little and mostly unchanged rates of immigration with the highest percentage of households living in social housing: ‘*Low and Stable—Social Housing’*. Cluster 3 is similar to this group in terms of low and mostly unchanged proportions of in‐movers but movers here are most likely to be 65 years old or older, and to own the property they live in: ‘*Low and Stable—Older Owning’*. Cluster 4 comprises wards with steeply increasing percentages of internal and international in‐movers to residents that are especially attractive to young movers: ‘*Increasing, Young Migration’*. Wards in cluster 5 show consistently high percentages of internal, and most significantly international, migrants in the population: ‘*High International Migration’*. Finally, Cluster 6 is the only group with decreasing proportions of in‐movers to residents, although the levels of migration remain high throughout: ‘*High but Declining Migration’*.

**Table 3:** Descriptive information of the clusters’ general population, area characteristics, and migration flows in 2011.

	1	2	3	4	5	6
Group	**‘**Steadily Increasing Migration’	**‘**Low and Stable— Social Housing’	‘Low and Stable— Older Owning’	**‘**Increasing, Young Migration’	**‘**High international Migration’	**‘**High but declining Migration’
% of wards accounted for	10.7	21.3	63.2	2.4	0.5	1.9
Population						
Total residents per cluster	9,687,389	15,607,590	32,593,672	2,149,507	381,170	951,987
Mean residents per ward	9965	7292	5076	10178	7619	5407
Population density^a^	105.86	64.08	36.39	130.16	183.96	63.48
Age						
% of population aged 0–18	19.46	22.32	20.14	13.11	16.03	20.58
% of population aged 18–24	29.56	21.00	15.98	66.43	30.45	18.01
% of population aged 35–59	20.00	24.17	29.58	8.13	20.61	26.94
% of population aged 65+	3.19	5.10	8.09	1.18	2.71	5.15
Employment status and SES						
% of adults that are students^b^	11.29	7.35	6.33	30.96	14.36	7.10
% of adults that are retired^c^	9.64	14.10	17.26	7.63	7.46	12.08
% of adults that are unemployed^d^	5.27	6.46	3.34	4.34	3.37	3.35
% of households that own one or more vehicles	60.80	65.15	83.50	54.16	51.73	77.79
Housing tenure						
% of households that own	48.87	54.31	74.78	37.49	38.32	59.16
% of households that socially rent	20.90	32.21	11.02	22.27	14.29	13.56
% of households that privately rent	27.88	11.61	12.24	37.92	42.83	24.46
Ethnicity						
% of population of White ethnicity	73.18	89.04	94.26	78.02	73.85	87.63
% of population of Black ethnicity	7.18	3.16	0.91	4.43	3.44	2.35
% of population of Asian ethnicity	13.92	5.40	3.18	12.24	12.30	5.77
% of population of Gypsy/Multiple/Other ethnicity	5.72	2.40	1.65	5.31	10.40	4.25
Household composition						
% of households with dependent children	27.19	30.98	28.41	18.42	22.21	29.51
% of adults that are single^e^	45.41	36.97	28.09	60.86	46.60	33.39
Migration						
% of population that moved within the ward since 2010	2.19	2.15	1.43	5.33	1.93	1.64
% of population that moved in from GB since 2010	13.17	7.04	7.32	25.77	17.42	10.93
% of population that moved in from abroad since 2010	2.22	0.57	.58	5.48	7.21	1.97
% of population that moved away to GB since 2010^f^	13.12	7.27	7.50	24.39	19.54	11.54
Internal net migration	0.68	−0.65	−0.49	11.13	−3.08	−1.30
Geography (% of wards in each cluster)						
Urban	97.46	89.58	66.59	98.58	89.26	64.21
In North East	2.16	10.17	3.28	6.28	0	1.14
In North West	7.01	12.83	9.88	11.11	2.0	1.7
In Yorkshire and the Humber	4.85	5.65	4.73	6.28	5.89	4.55
In East Midlands	5.67	6.35	10.3	7.73	1.9	6.25
In West Midlands	5.57	8.73	7.74	5.31	2.2	3.98
In East of England	8.66	6.21	13.2	4.35	14.61	19.89
In London	31.03	5.13	2.52	4.83	48.1	11.93
In South East	14.23	5.97	17.65	15.94	16.4	31.25
In South West	8.66	3.22	13.79	10.14	7.9	11.36
In Wales	3.81	10.22	9.14	9.18	0	2.84
In Scotland	8.35	25.52	7.76	18.84	2.1	5.11

*Note*: Differences between clusters in 2011 across a range of variables. Apart from ‘Percentage of wards accounted for’, ‘total usual residents’ and variables under ‘Geography’, cells show means across all cluster wards. ANOVA found all differences in means between the clusters to be statistically significant at the 0.5 level.

^a^ The number of usual residents per hectare.

^b^ Economically active and inactive students (including part‐time students) as a percentage of all usual residents aged 16–74.

^c^ Retirees as a percentage of all usual residents aged 16–74.

^d^ Those that unemployed but looking for a job, have never worked, or are long‐term unemployed as a percentage of all usual residents aged 16–74.

^e^ Those of marital status ‘single’ (never married or never registered a same‐sex civil partnership) as a percentage of all usual residents aged 16+.

^f^ The population base was calculated by adding the out‐movers and subtracting both international and internal in‐movers. With no data on international emigration, the true population base in 2010 may have been slightly larger than estimated.

#### 3.2.1 Steadily increasing migration

In total, 10.7% of wards in Great Britain belong to the *Steadily Increasing Migration* cluster. They are characterised by a moderate percentage of internal and international migrants in the population which has been increasing steadily since the 1980s. Movers are neither exceptionally old nor young with the balance between movers of different ages remaining fairly constant. Tenure trajectories also fall into the mid‐range. Apart from a slight drop in older and owner‐occupying in‐movers between 2001 and 2011, percentages of migrants in different age and tenure groups remain relatively stable over time. The additional descriptive information (Table 3) shows that this cluster has the lowest mean percentage of white residents in 2011 and, accordingly, the highest percentage of residents of Black and Asian ethnicity. Additionally, it is one of only two groups that maintains a positive net migration rate.

**Figure 2:**
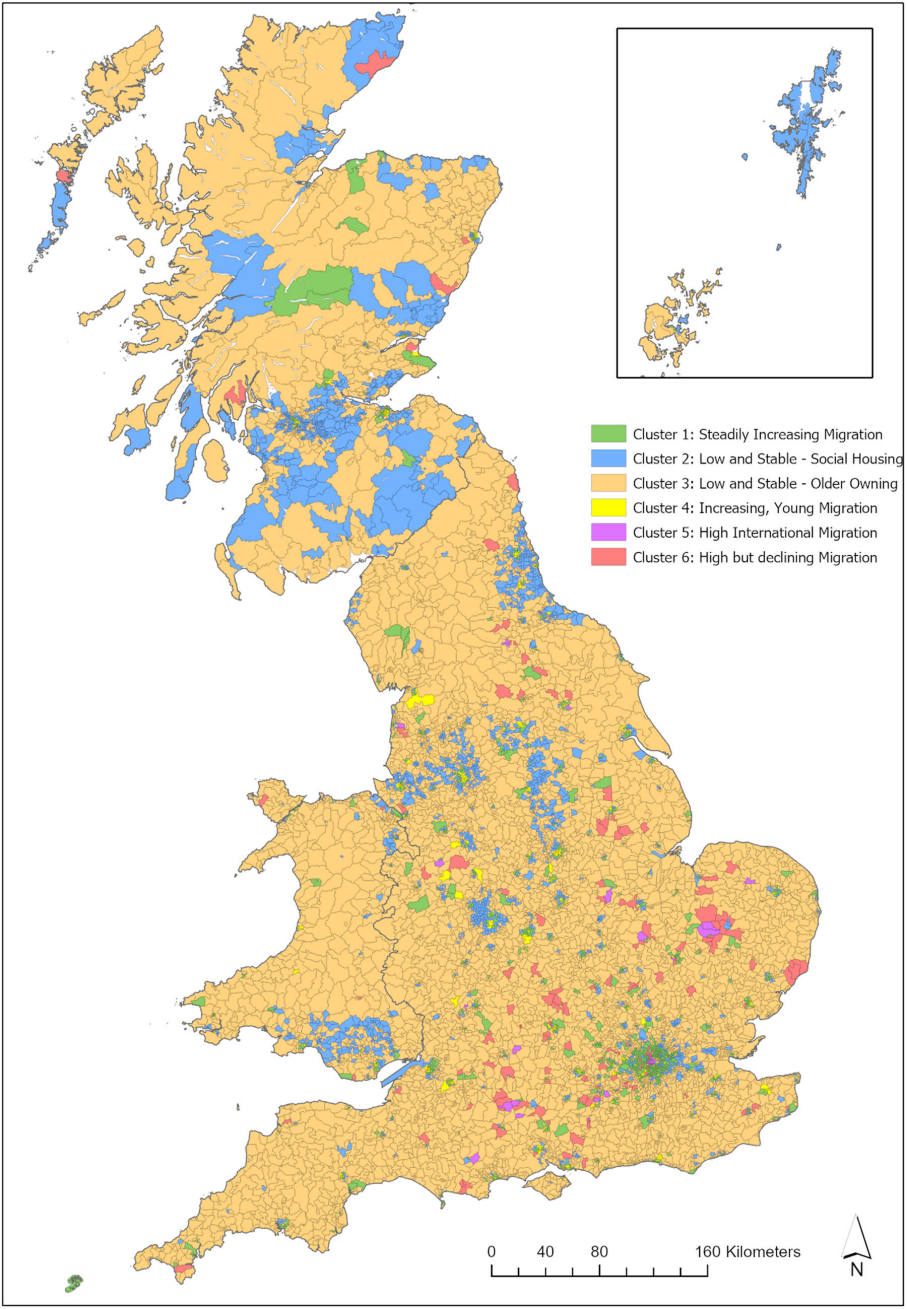
Cluster membership across Great Britain. Coloured map of Great Britain showing the cluster membership of wards. A map frame of the Orkneys and Shetlands is provided.

**Figure 3:**
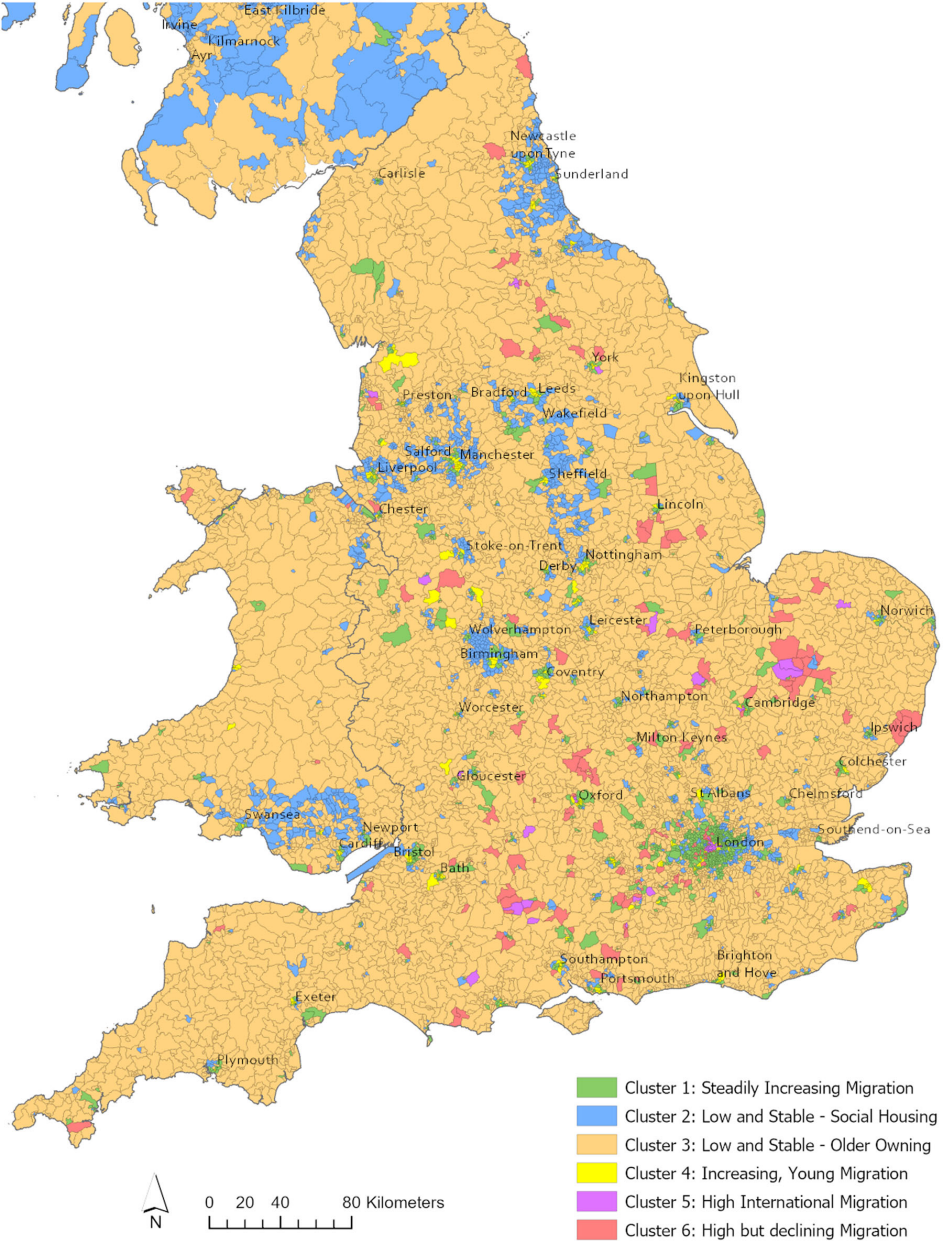
Cluster membership in England and Wales. Coloured map of England and Wales (mainland) showing the cluster membership of wards.

**Figure 4:**
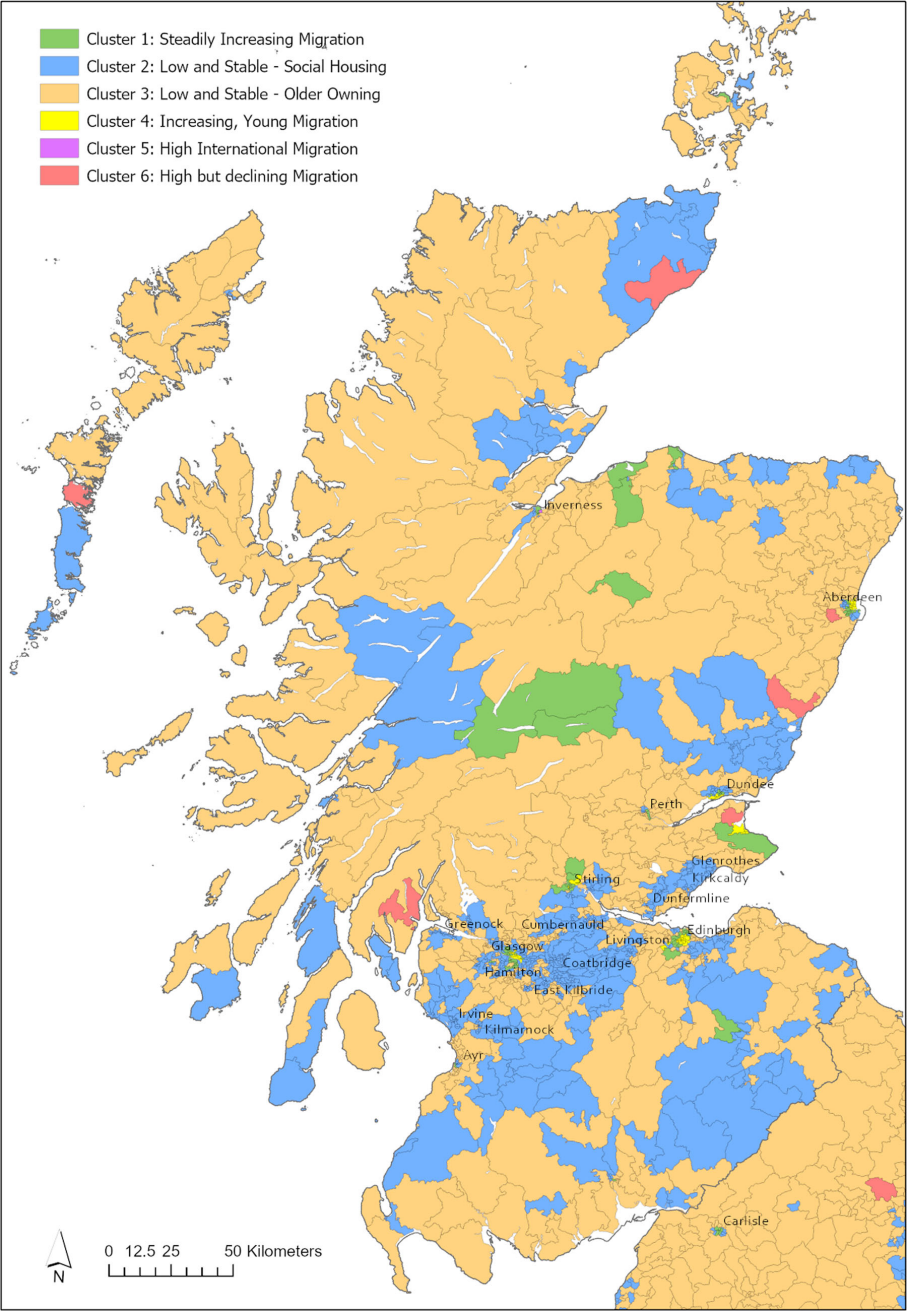
Cluster membership in mainland Scotland. Coloured map of mainland Scotland showing the cluster membership of wards.

Around 97% of wards in this cluster are characterised as urban, and this is also clearly visible when group‐membership is mapped (Figures 2, 3, 4). We see *Steadily Increasing Migration* wards in cities like Norwich, Coventry or Blackpool, and parts of Birmingham, Glasgow and London. Additionally, several *Steadily Increasing Migration* wards are located in coastal towns such as Eastbourne and Bournemouth, and parts of the Scottish highlands. The distribution across Government Office Regions (GORs) in Table 3 shows around a third of all *Steadily Increasing Migration* clusters are located in the London Region, followed by the South East. The lowest prevalence is in the North East.

#### 3.2.2 Low and stable—social housing

The *Low and Stable*—*Social Housing* cluster accounts for 21.3% of wards in Great Britain. It is characterised by low proportions of internal and international in‐movers to residents throughout the four time points. Additionally, in‐movers tend to be older with one of the highest percentages of movers over the age of 65, and lowest percentage of those between 18 and 24 years. Most outstanding for this cluster is the high percentage of in‐moving households living in social housing which sits above the other clusters at all time points. The descriptive information adds that in 2011, this cluster had the highest mean unemployment rate in its general population. Furthermore, the low percentage of in‐movers is mirrored by a low percentage of out‐movers; there is little turn‐over and the population remains mostly stable.

Geographically, wards of the *Low and Stable*—*Social Housing* cluster form multiple ward‐wide belts around larger cities. This is particularly true in the Midlands, towards the North East of England, and in the South Wales. The ‘belts’ are tight near the city core and become more dispersed and interrupted by other clusters moving outwards. A large number of Scottish wards fall into this cluster including areas near the Borders, parts of the Highlands and some of the Scottish islands (particularly the Shetlands and South Uist). Other Regions where the cluster is common are Wales, the North East and North West (with each GOR accounting for around 10% of *Low and Stable*—*Social Housing* wards).

#### 3.2.3 Low and stable—older owning

The *Low and Stable—Older Owning* cluster accounts for the largest percentage, around 63.2%, of wards in Great Britain. It is similar to the *Low and Stable—Social Housin*g cluster in terms of low and mostly unchanged proportions of internal and international movers across all four time points, and fairly low percentages of movers between the ages of 18 and 24. What distinguishes the *Low and Stable—Older Owning* cluster is that is has the highest percentage of movers over the age of 65 and the highest percentage of mover households who owner‐occupy. In contrast, the percentage of in‐mover households in social housing is one of the lowest. In 2011, the general population of this cluster was characterised by a higher percentage of retirees, and correspondingly, a lower percentage of students than any other cluster. Furthermore, residents of this cluster were predominantly white, around 84% owned one or more cars, and few were single. As the largest cluster in Great Britain, around 32 million residents lived in a *Low and Stable—Older Owning* ward in 2011.

Geographically, *Low and Stable*—*Older Owning* wards dominate the rural space in Great Britain and seldom reached into urban areas beyond the suburbs. The rural nature of this cluster is also demonstrated by the fact it had the lowest population density of all the clusters in 2011, with a mean of 35 people per hectare. Across the GOR, in 2011 this cluster is most commonly located in the South East, South West, and the East of England and is least commonly found in London.

#### 3.2.4 Increasing, young migration

In all, 2.4% of wards in Great Britain are classified as *Increasing, Young Migration*. The *Increasing, Young Migration* cluster is characterised by the highest and steeply increasing percentage of internal and international in‐movers among the population. Starting at an already high level, the percentage of in‐movers from Great Britain more than doubled between 1981 and 2011, and the percentage of international in‐movers rose by around 500%. Additionally, this cluster is noteworthy for its high and increasing percentage of movers between the ages of 18 and 24: nearly 70% of in‐movers in 2001 and 2011 in this age group. Correspondingly, the trajectory of movers over the age of 65 decreased over time. In terms of tenure, this cluster has moderate to low trajectories of owner‐occupying and socially‐renting mover households. The descriptive characteristics show that this cluster had the highest percentage of student residents in 2011. Additionally, higher than average proportions of residents were single, and relatively few had dependent children. The *Increasing, Young Migration* cluster had the highest internal net migration rate and the highest percentage of within ward movers.

Geographically, *Increasing, Young Migration* wards approximate the location of university town such as Oxford, Cambridge, Exeter, Bath, St. Andrews or Aberystwyth. Furthermore, they can be found in the cores of larger cities, such as Birmingham, Edinburgh and Manchester. The breakdown per GOR shows that *Increasing, Young Migration* wards are particularly common in Scotland and the South East, closely followed by the North East, North West and South West.

#### 3.2.5 High international migration

Accounting for only 0.5% of wards, the *High International Migration* Cluster stands out for having consistently high levels of in‐movers from Great Britain and abroad. The percentage of international migrants amongst residents exceeds that in the other cluster's trajectories by a wide margin across all four time points. At its peak in 2001, nearly 10% of residents in these wards had moved there from abroad within the previous year. Movers were neither exceptionally old nor young, but the trajectory for movers over the age of 65 years shows a slight increase over time (whilst it shows as decrease in most other clusters). In terms of tenure, the *High International Migration* cluster has consistently low trajectories for the percentages of mover households both owner‐occupying and socially renting. The descriptive information for the entire population in 2011 shows that *High International Migration* wards have low percentages of white residents and the highest percentages of ‘other’ or multiple ethnicities.

The small number of *High International Migration* wards is unevenly distributed across the GORs: around half are located in the London Region, and the remaining are predominantly in the South and East of England, mostly in smaller cities such as York, Oxford and Cambridge. The urban nature of this cluster is also demonstrated by the fact that it has highest population density with a mean of 183 residents per hectare. Wards belonging to this cluster also commonly appear as neighbours of *High but declining Migration* wards near American armed‐forces bases such as those near Amesbury, Croughton, and Mildenhall.

#### 3.2.6 High but declining Migration

Around 1.7% of wards in Great Britain are characterised as *High but declining Migration*. This is the only cluster experiencing a decrease in the proportions of in‐movers relative to residents, and this decrease is particularly steep for international movers. That being said, trajectories for both types of in‐mover remain comparably high and above the levels of the two ‘Low and Stable’ clusters at all four time points. Incoming movers fall into a mid‐age range, are moderately likely to owner‐occupy, and unlikely to live in social housing. The descriptive information shows that the general population in 2011 had high percentages of residents between the ages of 35 and 59 and with dependent children. Few were single. The internal net migration rate was the second lowest showing that the decreasing level of in‐movers was accompanied by a rising number of moves away from *High but declining Migration* wards.

As mentioned previously, this cluster is often located next to *High International Migration* wards in remote areas near foreign military bases. Additionally, *High but Declining Migration* wards appear on the outskirts of smaller cities, for example, near Blackpool, Peterborough and Aberdeen, and near the centre of London. Around a third of all wards in this cluster are located in the South East, with a low prevalence in the North East and North West.

### 3.3 Urban cluster geographies

**Figure 5:**
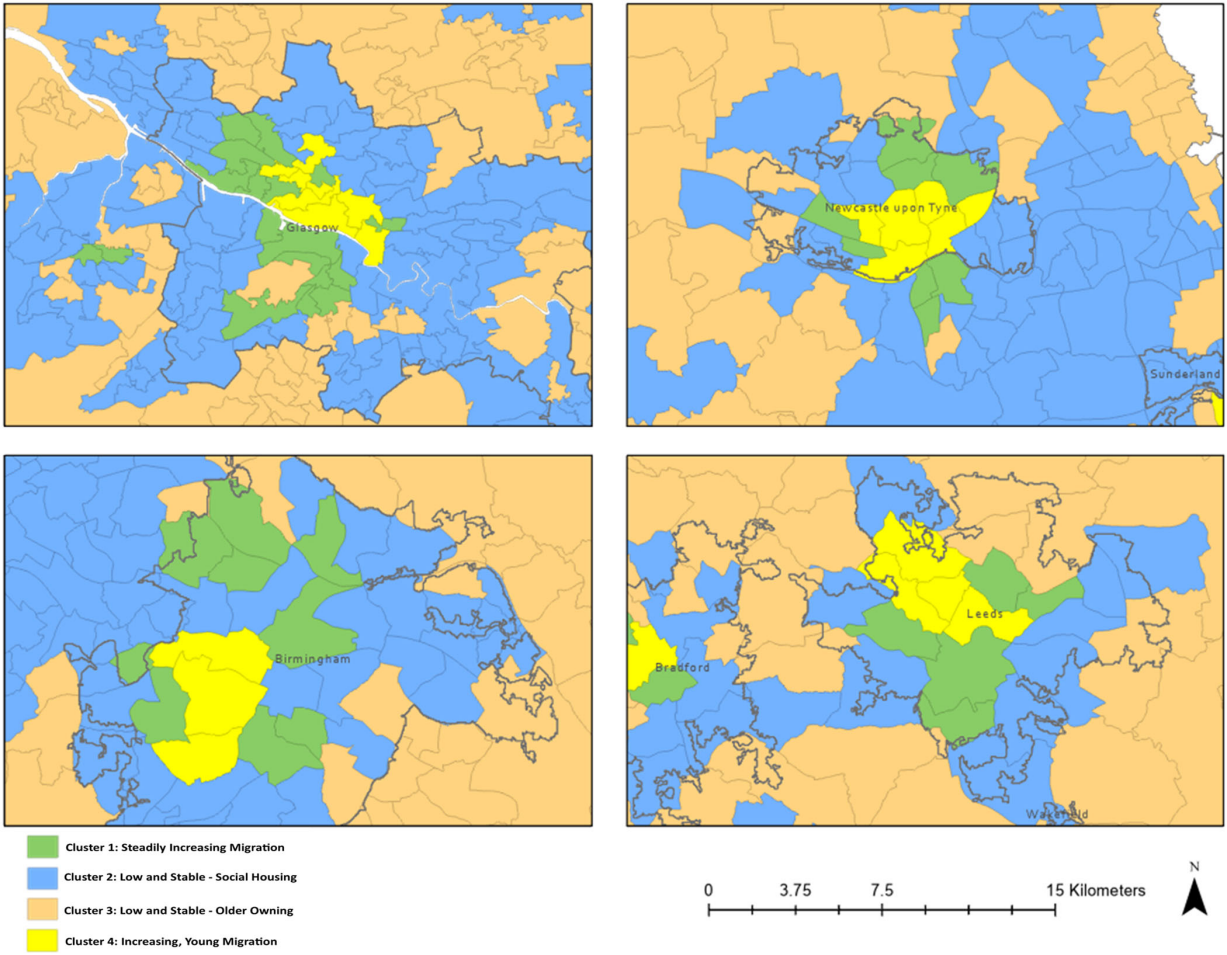
Cluster Distribution in Glasgow, Newcastle upon Tyne, Birmingham and Leeds**.** Coloured maps showing the cluster membership of wards in Glasgow, Newcastle upon Tyne, Birmingha, and Leeds.

**Figure 6:**
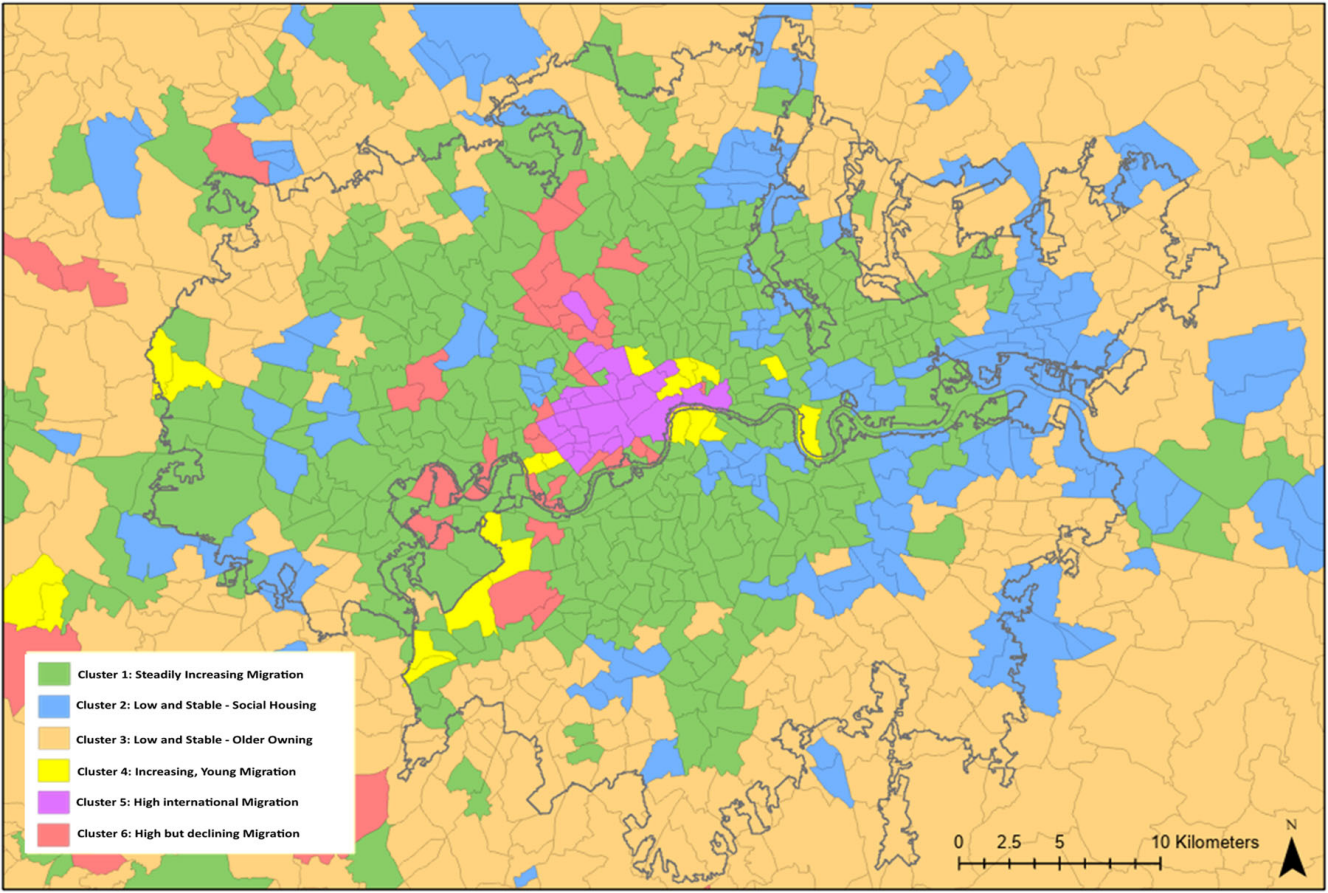
Cluster distribution in London. Coloured map of cluster membership in the city of London.

The geographical distribution of clusters forms a common pattern around many urban areas (Figure 5). Most cities consist of *Increasing, Young Migration* wards at their core, neighboured by *Steadily Increasing Migration* wards which are loosely bound by the *Low and Stable*—*Social Housing* cluster. Towards the outskirts of cities, the *Low and Stable*—*Social Housing* wards are broken up by *Low and Stable*—*Older Owning* wards which start to predominate outside the city boundaries. This pattern is reflected in the average distances between cluster centroids and city cores, which are 16, 71, 144 and 424 km, respectively. This pattern can be observed in large cities such as Birmingham, Manchester, Liverpool, Leeds, Nottingham, Newcastle upon Tyne, Edinburgh and Glasgow, and also smaller cities such as Exeter, Aberdeen and Portsmouth. Interestingly, however, this pattern cannot be observed in London (Figure 6). The London area shows a diverse and unique mix of clusters, and stands out for its high prevalence of *High International Migration* as well as *High but declining Migration* wards. Whereas most other city cores are dominated by *Increasing, Young Migration*, in London this role is taken by the *High International Migration* cluster. The majority of wards in London belong to the *Steadily Increasing Migration* cluster.

### 3.4 Comparison to existing area classifications

The main differences between our GBMTM classification and the ONS classification (2018) and the CIDER classification (Dennett & Stillwell, 2011) are that our classification considers changes through time, uses a different statistical approach to derive clusters and includes fewer variables. In addition, compared to the ONS classification, our classification includes information on those who move; and compared to the CIDER classification, our classification incorporates data on international migration.

Despite these differences, the chi‐square tests of independence indicate a significant relationship between our classification and the three versions of the ONS classification (2018) as well as the CIDER classification (Dennett & Stillwell, 2011) (Table 4). The size of the associations, measured using Cramer's V, sit at a moderate level.

**Table 4:** Comparisons and associations with existing area classifications.

Classification	ONS supergroup	ONS group	ONS subgroup	CIDER	Derived classification
Number of groups	8	26	76	8	6
Largest group^a^ (% of output areas)	‘Suburbanite’ (20.17%)	‘Semi‐Detached Suburbia’ (11.62%)	‘White Suburban Communities’ (4.25%)	‘Declining Industrial, Working‐Class, Local Britain’ (23.25%)	‘Low and Stable—Older Owning’ (51.00%)
Geographical scale^b^	Output areas	Output areas	Output areas	Districts	Standard table wards
Data source	2011 UK Census	2011 UK Census	2011 UK Census	2001 UK Census	1981, 1991, 2001 and 2011 UK Census
Derivation method	Hierarchical K‐means clustering	Hierarchical K‐means clustering	Hierarchical K‐means clustering	K‐means Cluster	GBMTM^c^
No. of variables used	59	59	59	56	6
Chi‐square test of association with the GBMTM classification					
Chi‐squared statistic	150,000	200,000	230,000	110,000	/
Degrees of freedom	35	125	375	35	/
*p* Value	<0.0001	<0.0001	<0.0001	<0.0001	/
Cramer's V	0.3595	0.4197	0.4498	0.312	/

*Note*:  The characteristics of the Office for National Statistics' 2011 general population‐based area classifications (Office for National Statistics, 2018) and Dennett and Stillwell's 2001 Migration‐centred CIDER Classification (Dennett & Stillwell, 2011) in comparison to the derived GBMTM classification. It includes the results of chi‐square tests of association between the existing classifications and the derived GBMTM classification.

^a^ Determined through the largest percentage share of output areas.

^b^ The ONS Classification is also available at the Local Authority level. To allow for comparison, all classifications were transformed to the lowest output area level before performing the test of association.

^c^ Group‐Based Multi‐Trajectory Modelling.

A closer look at the cross‐tabulation of the ONS classifications with our GBMTM clusters (Supporting Information: Appendix 2) revealed a noteworthy overlap between our *High International Migration* and their ‘EU White‐Collar Workers’ cluster, suggesting a largely European origin of the international in‐movers in this GBMTM cluster. Additionally, the ONS group ‘Hard‐Pressed Living’ corresponds moderately with the *Low and Stable—Social Housing* cluster. There is also a strong intersection between the ONS group ‘Cosmopolitans’ and our *Increasing, Young Migration* and the *High International Migration* clusters. Yet importantly, these two GBMTM clusters feature vastly different migration trajectories across the six variables, highlighting that our classification captures important differences within the ONS Cosmopolitan group that the ONS general purpose classification does not distinguish between. Similarly, some clusters, such as our *Steadily Increasing Migration* and *High but declining Migration* clusters, are evenly split across all ONS supergroups, indicating that the ONS classification does not account for their groups' distinct migration characteristics.

The cross‐tabulation with the CIDER classification (Appendix 2) shows a particularly high correspondence between our *Increasing, Young Migration* and their ‘Student Towns and Cities’, as well as our *High International Migration* and their ‘Dynamic London’ cluster. Notably, the ‘Dynamic London’ cluster also intersects with both our *Steadily Increasing Migration* and *High but declining Migration* clusters, indicating the presence of very diverse areas with distinct migration trajectories within the ‘Dynamic London’ cluster that are not captured by the CIDER classification. For instance, both the *Steadily Increasing Migration* and *High but declining Migration* cluster have similar percentages of internal movers amongst residents in 2001 (which is when the CIDER Classification collected their data), yet importantly, the former cluster exhibited consistent growth of in‐movers in the population over previous decades, while the latter experienced a decline. This disparity in change rather than the level of migration cannot be accurately represented by the cross‐sectional CIDER classification, emphasising the need for a cluster method capable of capturing temporal changes over time.

## 4 DISCUSSION

In this paper, we use GBMTM to generate a migration‐centred area classification for Great Britain based on six measures of migration and their development between 1981 and 2011. To our knowledge, this is the first study to classify longitudinal migration in the Great Britain and apply GBMTM to understand the flow of migration and its spatial form over time. The generated clusters highlight distinct geographical patterns in terms of number and type of movers they receive. Place characteristics are acknowledged as important determinants of migration (Norman et al., 2005; Walters, 2000), and our classification helps to outline what features have been important in ‘pulling’ different types of movers in recent decades, and where these are located spatially. In addition, it demonstrates that potential push or pull factors vary with age and stage in the life course. Similar to previous studies (Law & Warnes, 1976; Rogers, 1992), we found that young adults are drawn to (the centres of) urban areas, likely for education and employment opportunities, whereas moves in later life were predominantly to coastal or rural areas. Despite some clusters showing upward trajectories of in‐movers in relation to residents and positive migration rates in 2011, overall people in Great Britain are moving less which is in line with Champion and Shuttleworth's (2017a) analysis of migration within England and Wales between 1971 and 2011.

Area classifications identify patterns across different regions and help to understand their social, economic and demographic characteristics. The need for a migration specific classification is highlighted when our GBMTM classification is compared to the ONS general population classification (Office for National Statistics, 2018). Despite some general similarities, which provide validity to our findings and the method, the ONS classification does not distinguish different migration flows and often groups areas together that have different migration trajectories. For example, their general population cluster ‘Cosmopolitans’ strongly corresponds with our *Increasing, Young Migration* and the *High International Migration* cluster despite these two clusters having vastly different migration trajectories since the 1980s. Similarly, our *Steadily Increasing Migration* and *High but declining Migration* clusters are evenly split across all ONS supergroups, indicating that the general population clusters fail to account for the groups' distinct migration characteristics.

The GBMTM classification also has advantages over other migration‐specific classifications, because it adds a temporal perspective. The only other migration‐specific classification in the United Kingdom, the CIDER migration classification, is a static classification that does not account for changes in migration patterns over time. The importance of a longitudinal perspective in distinguishing migration trends is apparent in our *Steadily Increasing Migration* and *High but declining Migration* clusters. In 2001, the percentage of internal movers amongst residents is similar for both clusters, yet importantly, the former cluster experienced sustained, steady growth of in‐movers in the population in the previous decades, whereas the latter experienced a decline. The CIDER migration classification cannot account for this development and groups both clusters into the same ‘Dynamic London’ cluster. Whether migration is increasing or decreasing can have substantial effects on an area's age structure, economic efficiency, ethnic mix, housing market, labour demand, social capital, public goods and services, or even political participation (Akarca & Tansel, 2015; Bernt, 2019; Champion, 1999; Cooke, 2011; Crowder & South, 2005; Kaplan & Schulhofer‐Wohl, 2012; Lisenkova et al., 2014; van Dalen & Henkens, 2011). An accurate distinction between these migration developments is therefore critical for understanding the needs and challenges of areas and for making predictions. Furthermore, it provides the opportunity to analyse how migration patterns have evolved in response to economic or political events and to develop more effective policies to support those who move, those who stay, and those who live amongst newcomers.

Our classification aligns with several predominant migration trends. Processes of studentification are mirrored in the *Increasing, Young Migration* cluster, present in many city centres and typical student hubs such as Cambridge, Oxford, St. Andrews and Aberystwyth. *Increasing, Young Migration* wards show typical characteristics of student neighbourhoods with increased population churn, a large positive net migration rate (amongst young people), as well as increased inflow from abroad (Sage et al., 2012). Our finding that this cluster is geographically predominant in the South East of England is in line with over two decades of research emphasising this region as an ‘escalator’ for graduating students into employment (Duke‐Williams, 2009; Fielding, 1992; Findlay et al., 2009; Smith et al., 2016). The trajectories highlight the rapidity of population change and demographic restructuring that has occurred in these wards since 1981, but this might be partially attributable to the census accepting student term‐time addresses between 1991 and 2001. The observed speed of transformation is in line with earlier analyses by Dorling (1995) who described population change at the ward level for 1971–1981 and 1981–1991 showing increased mobility in areas of inner city student residence, and Sage et al. (2012) who investigated Elm Grove in Brighton (identified as *Increasing, Young Migration* in the current analysis) and advocated for the importance of longitudinal methodologies for correctly capturing urban change and migration. The fact that the percentage of student residents in this cluster far exceeds those of the other groups is consistent with previous ward‐level analyses by Bailey and Livingston (2005), who note that most wards in the United Kingdom have a student population of between 2% and 11%, but that studentifying wards can reach up to 61%.

A North–South divide is visible amongst the two largest clusters. In the *Low and Stable—Older Owning cluster* (widespread in the East and South of England) incomers predominantly purchase their home and in 2011 nearly 84% of the general population own at least one car. Stuart (2011) highlights that home‐owners in this part of the United Kingdom are in a strong economic position as high demand and high cost of housing has been consistently driving up the value of properties. In contrast, the *Low and Stable*—*Social Housing* cluster (accounting for nearly half of all wards in the North East and Scotland) has unemployment rates twice as high as the first and more than 30% of movers live in social housing at all investigated time points. On an assumption that tenure is a reflection of income, this suggests that movers into Northern regions were less affluent than those to the East or South. Additionally, the tenure characteristics in the general populations of these two clusters are similar to those of the in‐migrants, indicating that movers are not introducing new characteristics into these areas, but are sorting themselves into areas where they are similar to existing residents. This income selective migration can contribute to the persistence or widening of inequalities between the North and the South (Livingston & Bailey, 2007; Martikainen et al., 2008). Although both clusters show mostly low and stable proportions of in‐movers, Livingston and Bailey (2007) highlight that stability in turnover can carry different meanings and may reflect positive (satisfaction) or negative drives (dissatisfaction, but inability to move).

Other key patterns of recent British migration are urbanisation and counter‐urbanisation (A. G. Champion, 1989, 1994; T. Champion, 2005; Cross, 1990). As our classification is based on in‐migration and not net migration, its ability to reveal urbanisation and counter‐urbanisation trends is limited, as it is possible to have net out‐migration alongside a high and increasing percentages of in‐migrants in the population (as long as out‐migrants outweigh in‐migrants). In addition, our classification measures turn‐over rather than the stock of migrants and only considers migration flows since the 1980s. It is therefore possible that the classification misses some rural areas that have relatively high percentages of people who have ever‐migrated, but who then stay in the same place for a long time. We do, however, show that between 1981 and 2011 cities have stayed magnets for migrants, and that their populations are characterised by high turn‐over. This is opposite to the narratives of counter‐urbanisation that evoke images of stagnating and unpopular inner cities, coining phrases such as the ‘exodus from cities’. In addition, our analysis shows that many cities are made up of *Increasing, Young Migration* and *Steadily Increasing Migration* clusters which have a positive net migration rate in 2011, a possible sign of a resurgence of urbanisation. Similar patterns of returning urbanisation between 2001 and 2011 are found by Lomax et al. (2014) and Rae (2013). Yet, our ward‐level analysis also highlights that within the city boundary, some *Steadily Increasing Migration* are located more suburban than central, perhaps still representing a migration away from the city centre but not quite to the ‘rural idyll’. The importance of scale and possible deviations from overarching patterns of counter‐urbanisation at smaller geographies are emphasised by Dennett and Stillwell (2011) who, taking a district level approach, highlight that their ‘Young, vibrant cities’, ‘Regional centres’ and ‘Historic Centres’ clusters were all significant net gainers of population through migration in 2001.

Our classification shows a common geographical pattern of clusters in urban areas of different sizes and locations across Great Britain. Typically, city cores consist of *Increasing, Young Migration* and *Steadily Increasing Migration* wards, which are then loosely bound by *Low and Stable*—*Social Housing* and then *Low and Stable*—*Older Owning* wards. This shows that features of urban areas (e.g., the city centres or suburbs) act as pull‐factors of varying attractiveness to different population groups, thus engaging similar types and numbers of movers irrespective of the general size or location of a city. This varying ‘attractiveness’ is routed in different financial abilities, preferences, and needs, that vary throughout the life course (Champion et al., 1998; Law & Warnes, 1976; Rogers, 1992). Efforts to redirect or disperse these patterns through, for example, urban redevelopment projects may struggle to compete with these dynamics or, at least, should consider them as a substantial limitation. That cities have been experiencing recent migration in comparable ways, suggests there is opportunity to share experiences and solutions for (a) specific migration needs such as housing and infrastructure, or (b) challenges such as social cohesion and the construction of ‘student ghettos’ amongst different local actors.

London deviates from the observed urban pattern and shows a disorganised formation of clusters where wards in close proximity vary significantly in the type and numbers of migrants they attract. London's cluster formation could be unique to the capital given its distinctive history of population growth and ongoing global relevance in the labour market (Inwood, 2011; Schürer & Day, 2019), or it could be the product of sustained international and internal migration and related effects on the housing market. The latter suggests that other British cities could transition towards an increasingly mixed, London‐style of clustering once a certain period of time or population threshold is reached, a suggestion that fits into Geyer (1996) seven stage model of urbanisation. London's ‘super‐diversity’ (Vertovec, 2007), which mixes people with different religions, migration histories, ethnical, educational and economic backgrounds, can be seen as a positive process that fosters genuine exchange and a pride in ‘commonplace diversity’ (Wessendorf, 2013), or as a force to increase spatial polarisation and friction that maintains, if not increases, stereotyping and racism (Valentine, 2008; Watson, 2006) With potential effects on mental and physical health (Boyce et al., 2010; Mishra & Carleton, 2015). If cities are becoming increasingly mixed and diverse, it will be important to consider the resulting socio‐spatial inequalities and conflicts, and unpack the complex and intersecting ways in which difference is perceived and internalised.

### 4.1 Methodological reflections and limitations

To our knowledge, this study is the first application of GBMTM in the field of migration and one of the first to be applied to area rather than individual‐level data. A primary aim of this study was to test and reflect on the use of the method for this purpose. As it has been possible to produce groups and validate them through class profiling as well as comparisons to existing classifications, the model has achieved a satisfying outcome. The ability to consider multiple variables and their development over time provides many analytic opportunities that have thus far remained unexplored. That being said, there are several considerations and limitations to deliberate, some of which are unique to GBMTM, others are shared across all cluster analyses.

We have made a compelling case for the complexity of migration data and the need for broader approaches that encompass its multiple, longitudinal dimensions. The fact that GBMTM applications in Stata and SAS only allow for the inclusion of six indicators can therefore be considered a key constraint of the current analysis and the method's utility for migration studies. That being said, the recently released R‐package ‘gbmt’ integrates an Expectation‐Maximisation algorithm and offers the flexibility to include an unlimited number of variables, mitigating this constraint for future analyses. (Magrini, 2022b). To account for the small number of indicators in this study, we explored differences across a wider set of variables that were helpful to describe but less critical to define the clusters *after* cluster formation, allowing for a more detailed analysis and interpretation beyond just six indicators.

Another limitation of GBMTM is uncertainty and debate about the ideal class enumeration procedure (Klijn et al., 2017;  Nagin, 2005; Nagin & Odgers, 2010; Nagin et al., 2018). Selecting an appropriate number of clusters, and their parameter estimates, often remains subjective, particularly for multi‐trajectory models which need to be plausible on multiple measurement dimensions (Nagin, 2005). The balance between objective fit indices and subjective evaluation can be difficult to strike and introduces bias. The fact that GBMTM will not force a separation into clusters can be considered another limitation as it can encourage the exclusion of variables with ‘badly behaved distributions’ despite their potential significance to the analysis (D. Vickers & Rees, 2007), and may result in fewer groups and less granular results, hindering the scope for interpretation and further analysis. In the current study, only six groups could be distinguished and around 60% of wards fall into one cluster (the *Low and Stable—Older Owning*). However, the inability to force a separation into groups could also be considered a strength, as the model will only produce clusters that are supported by the data: if the data show only six main migration types and most of the rural land (which accounts for 90% of England's landmass; Scott, 2020) falls into one of them, then the conclusions that can be drawn from this are not necessarily limited but aligned with the truth.

The final set of limitations relates to challenges of suitable data inputs rather than the GBMTM technique itself. Variables must be comparable across time and space for a successful application of GBMTM to areas. However, in practice, area boundaries and measurement instruments can vary substantially through time. Existing approaches for linking data of incompatible areal units are often complex and require smaller area ‘bridges’ that are not always available (Martin et al., 2002). Some conversion in this paper had to therefore rely on lookup tables to make best‐fit assignments through areal weighting which ignored the relationship between migration and distance as well as uneven population distributions (Boyle & Feng, 2002). Missing indictors and measurement variation (including varying methods of disclosure control and degrees of data adjustments) are another limitation of data that, amongst others, led to the exclusion of Northern Ireland, inability to account for student term addresses and second homes, and use of broader age categories in this study. Measurement variation is also important for an extension of the classification to the most recent 2021/22 Census. Due to reporting during the Covid‐19 pandemic, which profoundly impacted people's ability to move home, migration trajectories will likely be distorted and the data discrepancy through Scotland's decision to delay their Census by 1 year will affect capacities to form comprehensive understandings of the entire U.K. migration system and to make sound comparisons (Lomax, 2022; Nanda et al., 2021). Until the 2021/22 Census migration data have been better understood it is not planned to extend the classification. Although alternative data sources such as the NHSCR or the Individual Sample of Anonymised Records (ISAR) may help to overcome some of these challenges, reliability and geographical coverage are unlikely to match that of the U.K. census (Rees et al., 2002; D. Vickers & Rees, 2007). Overall, data source and variable selection should be approached with care and (additional) procedures for reducing variability through time are imperative.

## 5 CONCLUSION

In this paper we present a novel application of GBMTM for creating a longitudinal, migration‐specific area classification that encompasses six indicators and their development between 1981 and 2011. The presented classification reveals six distinct migration clusters that are not sufficiently reflected in existing general purpose or cross‐sectional migration classifications. Our classification's longitudinal perspective distinguishes between migration trends and developments providing insights into, for example, the pace of studentification and persisting income‐selectivity in Northern and Southern areas. In addition, we show that cities have remained attractive to migrants between 1981 and 2011, contradicting counter‐urbanisation narratives suggesting stagnation and unpopularity in inner cities. The geographical distribution of clusters shows a common pattern in urban areas irrespective of size or location, indicating that certain urban features have been acting as common pull‐factors to different types of movers and that cities across Great Britain have been experiencing recent migration in comparable ways. Furthermore, many city cores contain clusters exhibiting a positive net migration rate in 2011, suggesting a potential resurgence of urbanization. Overall, GBMTM appears transferable to aggregate data and applications in the complex field of migration. Beyond accurately distinguishing between different developments of migration, the classification can help to identify variables for further analysis, such as the type, direction or pace of change. We conclude that GBMTM offers a thus far underexplored mean of succinctly summarising migration data over multiple time points that allows for a more accurate and comprehensive understanding of migration and its effects on local populations and areas. We believe that a longitudinal, migration‐focused perspective is critical to understand the complex temporal and spatial dimensions of migration, and that GBMTM provides a promising tool to achieve it.

## CONFLICT OF INTEREST STATEMENT

The authors declare no conflict of interest.

## Supporting information

Supporting information.

Supporting information.

## Data Availability

Data are available in public, open access repositories. The data underlying this article are freely available via the Nomis platform (https://www.nomisweb.co.uk/); CasWeb (https://casweb.ukdataservice.ac.uk/); or WICID (https://wicid.ukdataservice.ac.uk/).
