# Supplementary material for: A longitudinal area classification of migration in Great Britain: Testing the application of Group‐Based Multi‐Trajectory Modelling
Source: Popul Space Place. 2023 Jul 14;29(7):e2694. doi: 10.1002/psp.2694 (PMC10909560; doi:10.1002/psp.2694)
Supplement: Supplementary file 1 — Supporting information. [file PSP-29-e2694-s002.docx]

**Appendix 1: Methodological Appendix**

**Part 1: Area Conversions**

Approximately half of the data were available in 2001 ST wards (either as the official release or through the extensive, and officially recognized, conversion by Feng and Boyle (2002; 2010)). The remaining data were manually converted using areal weighting. An overview of the underpinning area conversions per indicator by year can be found below.

**Overview of indicators and area-boundary conversions**

| **Indicator** | **1981** | **1991** | **2001** | **2011** |
| --- | --- | --- | --- | --- |
| **International in-movers** | Feng and Boyle conversion;  NA for Scotland | Feng and Boyle conversion | Released in 2001 ST wards | Manually converted for England, Wales, and Scotland |
| **Internal in-movers** | Feng and Boyle conversion | Feng and Boyle conversion | Released in 2001 ST wards | Manually converted for England, Wales, and Scotland |
| **In-movers aged 18 – 24 years** | Manually converted for England, Wales, and Scotland | Feng and Boyle conversion | Released in 2001 ST wards | Manually converted for England, Wales, and Scotland |
| **In-movers aged 65 years and over** | Manually converted for England, Wales, and Scotland | Feng and Boyle conversion | Released in 2001 ST wards | Manually converted for England, Wales, and Scotland |
| **In-moving households who live in Social Housing** | NA | Feng and Boyle conversion for England and Wales;  Manually converted for Scotland | Released in 2001 ST wards | Manually converted for England, Wales, and Scotland |
| **In-moving households who owner occupy** | NA | Feng and Boyle conversion for England and Wales;  Manually converted for Scotland | Released in 2001 ST wards | Manually converted for England, Wales, and Scotland |
| **Usual Residents** | Manually converted for England, Wales, and Scotland | Manually converted for England, Wales, and Scotland | Manually converted for England, Wales, and Scotland | Manually converted for England, Wales, and Scotland |

In a few occasions, the manual conversions had to be based on conversion tables which only provided a general match between a source boundary and 2001 ST wards but not the exact percentage split. These overview tables also included the division into 2001 Output Areas. An excerpt of such a table can be found below:

| Source Boundary | 2001 ST Ward | 2001 Output Areas |
| --- | --- | --- |
| QAS31 | 01S28 | 60QA001405 |
| QAS31 | 01S21 | 60QA000014 |
| QAS32 | 01S28 | 60QA000015 |
| QAS32 | 01S28 | 60QA001468 |
| QAS32 | 01S20 | 60QA001467 |
| QAS32 | 01S21 | 60QA000016 |

To allow for conversion through such tables, it was assumed that Output Areas are comparable building blocks holding local populations of similar size in 2001. Accordingly, the data were divided by the number of Output Areas each source boundary was split into, and then added for each 2001 ST Ward. The calculations are outlined below:

| Source Boundary | e.g. Number of Movers | 2001 ST Ward | 2001 Output Areas | Calculation |
| --- | --- | --- | --- | --- |
| QAS31 | 50 | 01S28 | 60QA001405 | 50/2 |
| QAS31 | 50 | 01S21 | 60QA000014 | 50/2 |
| QAS32 | 100 | 01S28 | 60QA000015 | 100/4 |
| QAS32 | 100 | 01S28 | 60QA001468 | 100/4 |
| QAS32 | 100 | 01S20 | 60QA001467 | 100/4 |
| QAS32 | 100 | 01S21 | 60QA000016 | 100/4 |

01S28 = (50/2) + (100/4) + (100/4) = 75

01S21 = (50/2) + (100/4) = 50

01S20 = 100/4 = 25

To validate the manual conversions, the code was run on data for which a direct release in 2001 ST wards and also (a) a conversion table with percentage splits or (b) an overview table without the exact percentage split was available. Thereby, the outputs from the manual conversions could be compared to the official release and statistically tested for differences. Neither conversions that were based on conversions tables with percentage splits, nor conversions relying on the Output Area bridges were significantly different from the official releases with paired t-tests showing an average mean difference of -4.58% and p-value of 0.9999.

**References:**

Boyle, P., & Feng, Z. (2002). A method for integrating the 1981 and 1991 British census interaction data. *Computers, Environment and Urban Systems*, *26*(2), 241–256. https://doi.org/10.1016/S0198-9715(01)00043-6

Feng, Z., & Boyle, P. (2010). *Estimating Spatially Consistent Interaction Flows Across Three Censuses* [Chapter]. Technologies for Migration and Commuting Analysis: Spatial Interaction Data Applications. https://doi.org/10.4018/978-1-61520-755-8.ch013

**Part 2: Model Selection for GBMTM**

This section provides an extended description of the model selection process for the current study. For an all-encompassing overview of the method and underlying calculations please refer to Nagin’s ‘Group-based modelling of development’ (2005).

The selection process encompassed two main steps. First, the number of latent groups *k* was determined. For this, a maximum number of trajectory groups and a starting polynomial order describing the level and shape of each group’s trajectory graph, needed to be set. A polynomial order of 0 refers to a constant trajectory set at the intercept value, a polynomial order of 1 describes a linear trajectory, an order of 2 a quadratic trajectory and so forth. Both decisions can be arbitrary or theory driven but should consider the nature of the data as smaller samples may be overstretched if too many groups are fitted and fewer time points may prevent the use of higher order of polynomials. For this analysis, the maximum *k* was set as 7 and the order of polynomials were fixed at the highest order possible, meaning cubic for variables recorded at four time points and quadratic for those recorded at three time points.

In order to choose an appropriate number of latent groups, several models for different ‘ks' were run while keeping the order of polynomials constant, and observing the output from several statistical fit indices. A large number of goodness-of-fit and model adequacy indices were utilised in the current analysis: The Bayesian Information Criterion (BIC), the Akaike's Information Criteria (AIC), the Entropy, the Average Posterior Probability of Assignment (APPA), the Odds of Correct Classification (OCC), the mismatch between the estimated group probabilities and the proportion of the sample assigned to the group, and the percentage of individuals estimated to be assigned to the smallest group. An overview of the most frequently used fit indices and their recommended thresholds can be found in Klijn et al. (2017) and van der Nest et al. (2020). Additionally, models were evaluated on their substantive importance and ability to display the main trajectory paths that the analysis aims to highlight (this in turn was informed by univariate trajectory analysis of each indicator prior the main analysis).

The second step of model selection was concerned with adjusting the polynomial orders of trajectories. Keeping the selected k constant, polynomial terms were adjusted if significance tests of the polynomials terms in the model output returned p-values above 0.5 and/or the form of a given trajectory graph assimilated a different order shape. During these adjustments the BIC and AIC were closely observed and helped to decide on the best fitting model. Whilst group enumeration and the selection of polynomial orders are presented as two subsequent steps in this analysis, it is left to the researcher’s own discretion to follow this strict two-step approach or to conduct class enumeration and the tests of the regression coefficients simultaneously.

If a model cannot be fitted or results in errors, subsequent adjustments may be made to improve model fit. Adjustments can be made to the variables by, for example, setting outliers to the 99.9th percentile value, or to the model by manually revising the matrix of starting values that specifies where the model should start searching for the Maximum Likelihood solution. Such a matrix of starting values can be informed by the data directly or by running more simplified models, either by reducing the number of groups or the number of variables, and slowly building up a matrix from the output parameter estimates. A schematic overview of the different analysis steps is provided below (Figure 1).

**Figure 1:** **Analysis plan**


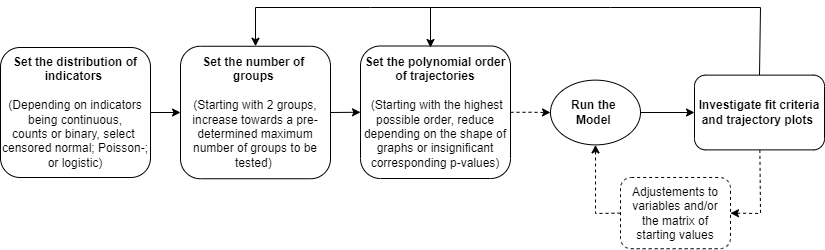


**References:**

Klijn, S. L., Weijenberg, M. P., Lemmens, P., van den Brandt, P. A., & Lima Passos, V. (2017). Introducing the fit-criteria assessment plot – A visualisation tool to assist class enumeration in group-based trajectory modelling. *Statistical Methods in Medical Research*, *26*(5), 2424–2436. https://doi.org/10.1177/0962280215598665

Nagin, D. (2005). *Group-Based Modeling of Development*. Harvard University Press. https://www.degruyter.com/document/doi/10.4159/9780674041318/html

van der Nest, G., Lima Passos, V., Candel, M. J. J. M., & van Breukelen, G. J. P. (2020). An overview of mixture modelling for latent evolutions in longitudinal data: Modelling approaches, fit statistics and software. *Advances in Life Course Research*, *43*, 100323. https://doi.org/10.1016/j.alcr.2019.100323
