# Supplementary material for: A longitudinal area classification of migration in Great Britain: Testing the application of Group‐Based Multi‐Trajectory Modelling
Source: Popul Space Place. 2023 Jul 14;29(7):e2694. doi: 10.1002/psp.2694 (PMC10909560; doi:10.1002/psp.2694)
Supplement: Supplementary file 2 — Supporting information. [file PSP-29-e2694-s001.docx]

**Appendix 2: Comparison to existing area- classifications**

The table below shows the percentage split of the derived cluster across the three tiers of the general purpose classification by the ONS (2018); and the migration-specific CIDER classification (Dennett & Stillwell, 2011). Green shading indicates the size of overlap from light (little overlap) to dark (strong overlap).

**Table: Cross-Tabulation with existing area-classifications**

|  | **Steadily Increasing Migration** | **Low and Stable – Social Housing** | **Low and Stable – Older Owning** | **Increasing, Young Migration** | **High International Migration** | **High but declining Migration** |
| --- | --- | --- | --- | --- | --- | --- |
| **ONS Supergroups** |  |  |  |  |  |  |
| Constrained City Dwellers | 8.9 | 24.9 | 6.1 | 6.3 | 1.4 | 2.2 |
| Cosmopolitans | 16.1 | 1 | 0.7 | 50.7 | 58.3 | 17.9 |
| Ethnicity Central | 20.4 | 3.8 | 0.4 | 12.3 | 18.8 | 10.9 |
| Hard-Pressed Living | 4.8 | 34.8 | 15.8 | 2 | 2.2 | 7.1 |
| Multicultural Metropolitans | 26.2 | 11.8 | 4.3 | 15.5 | 3.5 | 9.1 |
| Rural Residents | 1.4 | 3.6 | 19.6 | 0.9 | 5.3 | 17.3 |
| Suburbanities | 5.4 | 11.4 | 31.3 | 3.2 | 1.8 | 11.6 |
| Urbanities | 16.8 | 8.6 | 21.7 | 9 | 8.6 | 23.9 |
|  | 100 | 100 | 100 | 100 | 100 | 100 |
|  |  |  |  |  |  |  |
| **ONS Groups** |  |  |  |  |  |  |
| Farming Communities | 0.5 | 1.4 | 7.1 | 0.4 | 1.7 | 4.9 |
| Rural Tenants | 0.7 | 1.8 | 10 | 0.4 | 3 | 10.2 |
| Ageing Rural Dwellers | 0.2 | 0.4 | 2.6 | 0.2 | 0.6 | 2.1 |
| Students Around Campus | 1.6 | 0.1 | 0.1 | 20.19 | 4.6 | 0.6 |
| Inner-City Students | 2.1 | 1.2 | 0.1 | 18.7 | 8.1 | 0.6 |
| Comfortable Cosmopolitans | 6.8 | 0.7 | 0.6 | 8.7 | 0.2 | 0.3 |
| Aspiring and Affluent | 5.6 | 0.1 | 0.1 | 3.1 | 45.4 | 16.4 |
| Ethnic Family Life | 5.6 | 1.5 | 0.1 | 4.3 | 0.4 | 1.3 |
| Endeavouring Ethnic Mix | 5.2 | 8.8 | 0.1 | 3.4 | 12.2 | 3.4 |
| Ethnic Dynamics | 1.5 | 1.1 | 0.1 | 3.2 | 0.5 | 0.1 |
| Aspirational Techies | 8.1 | 0.5 | 0.2 | 1.4 | 5.8 | 6.2 |
| Rented Family Living | 9.8 | 6.9 | 2 | 7.3 | 2.4 | 3 |
| Challenged Asian Terraces | 8.3 | 3.3 | 0.5 | 5.4 | 0.2 | 0.4 |
| Asian Traits | 8.1 | 1.6 | 1.8 | 2.8 | 0.9 | 5.7 |
| Urban Professionals and Families | 10.7 | 5 | 11.2 | 4.5 | 5.9 | 14.1 |
| Ageing Urban Living | 6.1 | 2.6 | 10.5 | 4.5 | 2.8 | 9.8 |
| Suburban Achievers | 2.7 | 3.2 | 14 | 2.1 | 0.6 | 6.6 |
| Semi-Detached Suburbia | 2.7 | 8.2 | 17.4 | 1.1 | 1.3 | 5 |
| Challenged Diversity | 5 | 7.2 | 2.2 | 2.7 | 0.7 | 1.4 |
| Constrained Flat Dwellers | 1.8 | 5.2 | 0.5 | 2.1 | 0.2 | 0.1 |
| White Communities | 0.9 | 10.4 | 2 | 0.5 | 0.4 | 0.4 |
| Ageing City Dwellers | 1.3 | 2 | 1.4 | 1 | 0.1 | 0.4 |
| Industrious Communities | 0.9 | 7.6 | 5.1 | 0.2 | 0.7 | 2.5 |
| Challenged Terraced Workers | 1.2 | 8.4 | 2.6 | 0.3 | 0.3 | 0.4 |
| Hard-Pressed Ageing Workers | 1 | 7.9 | 6 | 0.3 | 0.6 | 2.4 |
| Migration and Churn | 1.8 | 10.9 | 2.1 | 1.3 | 0.6 | 1.7 |
|  | 100 | 100 | 100 | 100 | 100 | 100 |
|  |  |  |  |  |  |  |
| **ONS Subgroups** |  |  |  |  |  |  |
| Rural Workers and Families | 0.05 | 0.49 | 1.28 | 0.04 | 0.24 | 0.78 |
| Established Farming Communities | 0.14 | 0.2 | 2.37 | 0.14 | 0.78 | 1.83 |
| Agricultural Communities | 0.15 | 0.5 | 2.26 | 0.1 | 0.31 | 1.61 |
| Older Farming Communities | 0.12 | 0.22 | 1.18 | 0.11 | 0.31 | 0.69 |
| Rural Life | 0.21 | 0.81 | 3.64 | 0.18 | 1.49 | 3.92 |
| Rural White-Collar Workers | 0.28 | 0.27 | 3.73 | 0.12 | 1.26 | 4.86 |
| Ageing Rural Flat Tenants | 0.2 | 0.69 | 2.58 | 0.09 | 0.31 | 1.44 |
| Rural Employment and Retirees | 0.14 | 0.12 | 0.74 | 0.08 | 0.39 | 1 |
| Renting Rural Retirement | 0.05 | 0.2 | 0.86 | 0.01 | 0.08 | 0.72 |
| Detached Rural Retirement | 0.07 | 0.12 | 0.94 | 0.09 | 0.08 | 0.42 |
| Student Communal Living | 0.24 | 0.01 | 0.01 | 3.61 | 2.59 | 0.17 |
| Student Digs | 0.06 | 0.01 | 0 | 5.81 | 0 | 0 |
| Students and Professionals | 1.3 | 0.02 | 0.01 | 10.77 | 2.04 | 0.44 |
| Students and Commuters | 0.94 | 0.07 | 0 | 11.81 | 0 | 0.03 |
| Multicultural Student Neighbourhoods | 1.12 | 0.11 | 0 | 6.88 | 8.08 | 0.61 |
| Migrant Families | 2.81 | 0.24 | 0.33 | 1.63 | 0.08 | 0.22 |
| Migrant Commuters | 1.91 | 0.29 | 0.06 | 2.88 | 0 | 0 |
| Professional Service Cosmopolitans | 2.09 | 0.16 | 0.16 | 4.16 | 0.16 | 0.06 |
| Urban Cultural Mix | 1.58 | 0.04 | 0.12 | 1.09 | 3.38 | 4.05 |
| Highly-Qualified Quaternary Workers | 2.62 | 0.02 | 0.02 | 1.05 | 10.68 | 5.55 |
| EU White-Collar Workers | 1.4 | 0.02 | 0 | 0.99 | 31.32 | 6.8 |
| Established Renting Families | 2.57 | 1.18 | 0.09 | 1.99 | 0.08 | 0.42 |
| Young Families and Students | 2.98 | 0.33 | 0.04 | 2.26 | 0.31 | 0.83 |
| Striving Service Workers | 2.51 | 0.56 | 0.01 | 0.71 | 1.26 | 1.39 |
| Bangladeshi Mixed Employment | 1.21 | 0.14 | 0 | 1.05 | 2.83 | 0.47 |
| Multi-Ethnic Professional Service Workers | 1.5 | 0.12 | 0.01 | 1.66 | 8.08 | 1.53 |
| Constrained Neighbourhoods | 1.11 | 0.57 | 0.06 | 2.27 | 0.47 | 0.08 |
| Constrained Commuters | 0.39 | 0.49 | 0.01 | 0.95 | 0 | 0 |
| New EU Tech Workers | 2.24 | 0.07 | 0.1 | 0.35 | 1.18 | 2.83 |
| Established Tech Workers | 3.1 | 0.25 | 0.1 | 0.27 | 0.47 | 0.89 |
| Old EU Tech Workers | 2.75 | 0.15 | 0 | 0.76 | 4.16 | 2.44 |
| Private Renting Young Families | 2.38 | 4.12 | 0.7 | 2.38 | 0 | 0.39 |
| Social Renting New Arrivals | 4.87 | 1.04 | 0.56 | 3.98 | 2.43 | 1.83 |
| Commuters with Young Families | 2.61 | 1.73 | 0.71 | 0.92 | 0 | 0.78 |
| Asian Terraces and Flats | 6.17 | 1.17 | 0.22 | 4.03 | 0.24 | 0.31 |
| Pakistani Communities | 2.09 | 2.17 | 0.32 | 1.33 | 0 | 0.06 |
| Achieving Minorities | 1.85 | 0.92 | 0.85 | 0.88 | 0 | 0.75 |
| Multicultural New Arrivals | 3.21 | 0.35 | 0.34 | 0.17 | 0.08 | 1.19 |
| Inner City Ethnic Mix | 3.02 | 0.27 | 0.65 | 1.77 | 0.78 | 3.75 |
| White Professionals | 1.67 | 2.05 | 5.08 | 0.6 | 0.39 | 3.75 |
| Multi-Ethnic Professionals with Families | 3.21 | 1.49 | 3.38 | 1.46 | 3.22 | 6.19 |
| Families in Terraces and Flats | 5.85 | 1.49 | 2.79 | 2.48 | 2.28 | 4.19 |
| Delayed Retirement | 2.6 | 0.76 | 2.72 | 2.58 | 1.18 | 4.03 |
| Communal Retirement | 1.84 | 0.7 | 2.38 | 1.05 | 0.94 | 2.22 |
| Self-Sufficient Retirement | 1.63 | 2.09 | 5.41 | 0.86 | 0.63 | 3.55 |
| Indian Tech Achievers | 1.05 | 0.52 | 2.2 | 0.85 | 0 | 2.42 |
| Comfortable Suburbia | 0.26 | 1.05 | 2.98 | 0.04 | 0.08 | 1.14 |
| Detached Retirement Living | 0.51 | 1.04 | 5.32 | 0.39 | 0.31 | 1.75 |
| Ageing in Suburbia | 0.88 | 0.59 | 3.44 | 0.82 | 0.16 | 1.31 |
| Multi-Ethnic Suburbia | 0.84 | 1.12 | 2.04 | 0.4 | 0.08 | 0.22 |
| White Suburban Communities | 0.73 | 2.49 | 6.68 | 0.27 | 0.55 | 3.28 |
| Semi-Detached Ageing | 0.64 | 2.75 | 5.14 | 0.27 | 0.24 | 0.75 |
| Older Workers and Retirement | 0.53 | 1.88 | 3.5 | 0.2 | 0.39 | 0.78 |
| Transitional Eastern European Neighbourhoods | 1.29 | 1.65 | 0.43 | 0.39 | 0.39 | 0.36 |
| Hampered Aspiration | 2.57 | 1.45 | 1.02 | 0.9 | 0.24 | 0.53 |
| Multi-Ethnic Hardship | 1.13 | 4.11 | 0.73 | 1.36 | 0.08 | 0.5 |
| Eastern European Communities | 0.55 | 1.54 | 0.07 | 0.94 | 0.24 | 0 |
| Deprived Neighbourhoods | 0.39 | 1.84 | 0.06 | 0.57 | 0 | 0 |
| Endeavouring Flat Dwellers | 0.86 | 1.86 | 0.38 | 0.61 | 0 | 0.03 |
| Challenged Transitionaries | 0.35 | 3.65 | 0.71 | 0.19 | 0.16 | 0.06 |
| Constrained Young Families | 0.25 | 4.08 | 0.35 | 0.18 | 0.16 | 0.06 |
| Outer City Hardship | 0.24 | 2.69 | 0.92 | 0.14 | 0.08 | 0.31 |
| Ageing Communities and Families | 0.44 | 0.67 | 0.78 | 0.31 | 0.08 | 0.25 |
| Retired Independent City Dwellers | 0.45 | 0.69 | 0.23 | 0.39 | 0 | 0.06 |
| Retired Communal City Dwellers | 0.22 | 0.44 | 0.31 | 0.1 | 0 | 0.08 |
| Retired City Hardship | 0.14 | 0.2 | 0.1 | 0.22 | 0 | 0 |
| Industrious Transitions | 0.62 | 3.31 | 3.32 | 0.12 | 0.31 | 1.97 |
| Industrious Hardship | 0.26 | 4.31 | 1.78 | 0.04 | 0.39 | 0.56 |
| Deprived Blue-Collar Terraces | 0.97 | 2.99 | 1.66 | 0.17 | 0.16 | 0.31 |
| Hard-Pressed Rented Terraces | 0.24 | 5.45 | 0.93 | 0.11 | 0.16 | 0.06 |
| Ageing Industrious Workers | 0.39 | 2.81 | 2.99 | 0.16 | 0.47 | 1.25 |
| Ageing Rural Industry Workers | 0.33 | 1.48 | 1.69 | 0.09 | 0.08 | 0.97 |
| Renting Hard-Pressed Workers | 0.24 | 3.67 | 1.31 | 0.06 | 0.08 | 0.22 |
| Young Hard-Pressed Families | 0.24 | 4.31 | 0.45 | 0.32 | 0 | 0.25 |
| Hard-Pressed Ethnic Mix | 0.78 | 4.31 | 1.15 | 0.66 | 0.24 | 0.56 |
| Hard-Pressed European Settlers | 0.78 | 4.31 | 1.15 | 0.66 | 0.24 | 0.56 |
|  | 100 | 100 | 100 | 100 | 100 | 100 |
| **CIDER Classification** |  |  |  |  |  |  |
| Coastal and Rural Retirement Migrants | 7.63 | 9.51 | 18.72 | 0.47 | 5.12 | 9.44 |
| Declining Industrial, Working-Class, Local Britain | 5.8 | 43.46 | 19.75 | 2.76 | 0 | 2 |
| Dynamic London | 34.31 | 4.37 | 2.49 | 10.33 | 80.03 | 38.4 |
| Footloose, Middle-Class, Commuter Britain | 6.8 | 1.81 | 12.66 | 2.15 | 8.09 | 22 |
| Low-Mobility Britain | 0.66 | 6.61 | 11.39 | 1.07 | 0 | 1.17 |
| Moderate Mobility, Non-Household, Mixed Occupations | 10.98 | 6.27 | 9.46 | 1.98 | 0.83 | 10.2 |
| Student Towns and Cities | 31.88 | 25.67 | 10.75 | 81.24 | 3.47 | 4.57 |
| Successful Family In-Migrants | 1.98 | 2.3 | 14.78 | 0 | 2.48 | 12.1 |
|  | 100 | 100 | 100 | 100 | 100 | 100 |
